# Effects of eye closure on the spiking activity of human lateral geniculate neurons

**DOI:** 10.1038/s41467-025-65383-x

**Published:** 2025-11-24

**Authors:** Matthew W. Self, Osvaldo Vilela-Filho, Sergio Neuenschwander, Hélio F. Silva-Filho, Lissa C. Goulart, Pieter R. Roelfsema

**Affiliations:** 1 Department of Vision & Cognition, Netherlands Institute for Neuroscience, Amsterdam, The Netherlands; 2 School of Psychology and Neuroscience, 62 Hillhead Street, University of Glasgow, Glasgow, Scotland; 3 Division of Neurosurgery, Department of Surgery, Medical School, Federal University of Goiás, Goiânia, Brazil; 4 Departments of Neurosurgery and Neurology, Clinics Hospital, Federal University of Goiás, Goiânia, Brazil; 5 Brain Institute, Federal University of Rio Grande do Norte, Natal, Brazil; 6 Department of Integrative Neurophysiology, Center for Neurogenomics and Cognitive Research, VU University, Amsterdam, The Netherlands; 7 Neurosurgery Department, Academic University Medical Center, Amsterdam, The Netherlands; 8 Laboratory of Visual Brain Therapy, Sorbonne Université, Institut National de la Santé et de la Recherche Médicale, Centre National de la Recherche Scientifique, Institut de la Vision, Paris, France

**Keywords:** Sensory processing, Thalamus

## Abstract

The lateral geniculate nucleus (LGN) of the thalamus is a key link between the retina and visual cortex but our understanding of the properties of neurons in the human LGN is based on recordings in animal models. Here we recorded spiking activity of cells in the LGN of two patients who had electrodes implanted in the LGN as part of their treatment for epilepsy. Human LGN cells responded to strong visual stimulation with high-frequency bursts of spikes. The cells had receptive-field properties resembling those of monkeys with circular ON-OFF sub-fields, red-green opponency in the dorsal layers and preferences for high temporal frequencies in the ventral layers. Responses were largely monocular and the closure of one eye decreased the spontaneous activity of broad-spiking neurons preferring this eye while increasing the activity of neurons with narrower spikes, suggesting that interneurons might gate LGN activity during eye closure.

## Abstract (web summary)

The LGN is a critical stage between the retina and visual cortex, but the properties of human LGN neurons are not fully understood. Here the authors report that they closely resemble those in monkeys and that closure of one eye increases the activity of putative inhibitory neurons connected to that eye.

## Introduction

The dorsal lateral geniculate nucleus (LGN) is the main target of the axons of retinal ganglion cells in humans where these cells synapse with geniculate relay cells. Relay cells send their axons in turn via the optic radiation predominantly to the primary visual cortex (V1). The LGN is therefore a critical processing stage in the visual system and damaging it may cause scotomas and hemianopia^1^. LGN neurons have been studied extensively in cats and primates, detailing the laminar organization of the nucleus and the functional organization of receptive-field properties^2–5^. So far, research in humans has been limited to structural and functional magnetic resonance imaging (fMRI) studies, and it remains an open question to what extent the properties of single neurons in the human LGN resemble those in other species. Here we describe a rare surgical approach in which Deep Brain Stimulation (DBS) electrodes were placed in the LGN of two epileptic patients. The LGN was first mapped with high-impedance electrodes while the patients were awake, allowing the recording of spiking activity at multiple depths in the LGN. We investigated the similarity of human LGN neurons to those of other species, including primates.

Early studies found that the receptive field (RF) and tuning properties of LGN neurons of cats and monkeys resembled those of retinal ganglion cells. LGN neurons have concentric ON/OFF subfields^2–4^ which are well modeled by a linear difference-of Gaussians function^6^. In most primates, the LGN has six layers. The innermost, ventral two layers contain magnocellular neurons that are specialized for motion processing: they have high contrast sensitivity, prefer low spatial and high temporal frequencies and they have weak color tuning. The outermost, dorsal four layers contain red-green opponent parvocellular neurons that are specialized for high-resolution vision^5^, preferring higher spatial frequencies and lower temporal frequencies than magnocellular neurons. A third cell class is formed by koniocellular neurons in the cell-sparse regions between the layers, which process S-cone inputs that signal blue-yellow contrast^7^ as well as smaller numbers of cells with more diverse tuning properties. It remains unknown whether cells in the human LGN share these response characteristics, and we therefore investigated the color preferences and spatial- and temporal-frequency tuning profiles of neurons in the human LGN.

Each layer of the LGN receives inputs largely from one eye^3,4,8^; layers 1,4 and 6 from the contralateral eye, and layers 2, 3, and 5 from the ipsilateral eye. The responses of LGN neurons in cats and monkeys are, however, not purely monocular^9^. Stimulation of the non-dominant eye reduces the response of many cells to a stimulus in the dominant eye^10–12^, and there also is a smaller fraction of neurons that exhibit binocular facilitation^11^. Binocular modulation of responses is thought to be weaker in primates than in the cat^10,12,13^, raising the question of whether binocular interactions exist in human LGN. Human fMRI experiments suggest strong inhibitory interactions between cells representing each eye^14^, but it is unclear whether these signals reflect the activity of corticogeniculate inputs or activity intrinsic to the LGN^15^. Furthermore, humans can voluntarily close one eye, halving the input to the visual system, while experiencing only minor changes in visual perception. This form of gain control is thought to involve competition between the representations of each eye at both monocular and binocular stages of visual processing, but the neural mechanisms remain unclear. We therefore also investigated the eye preferences and the binocular interactions of individual neurons in the human LGN and examined the effects of closing one eye.

## Results

**Fig. 1:**
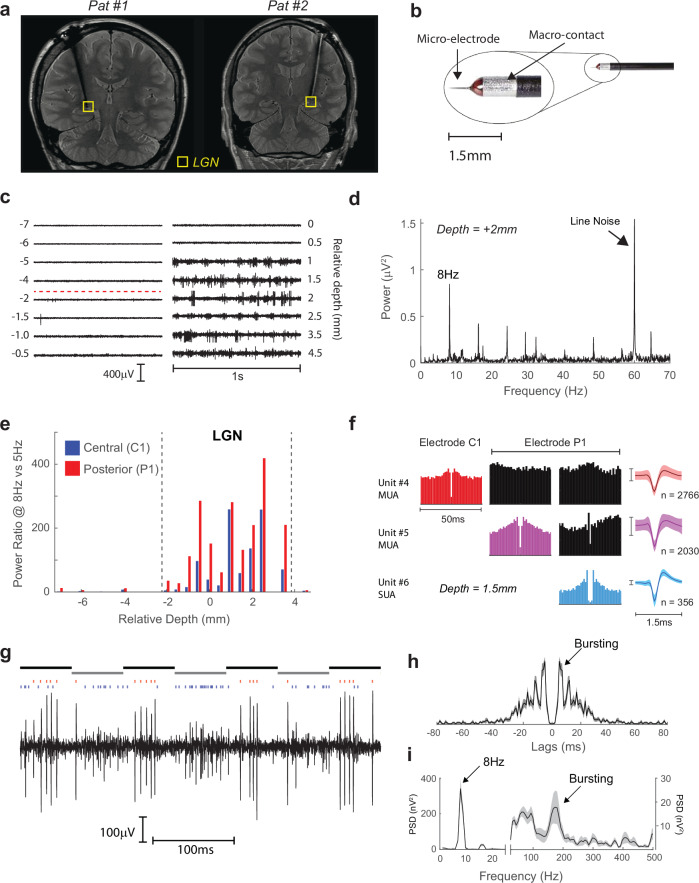
Recordings of single LGN neurons. **a** Postoperative coronal MR images from Patient #1 (image used with permission from reference #16) and Patient #2. The electrode trajectory targeted the LGN (yellow box). **b** The probe was comprised of a high impedance (0.8–0.9 MΩ) microelectrode and a macroelectrode for stimulation (Image courtesy of InoMed Neurocare Ltd.). Two microelectrodes were used in each patient to map spiking activity on a trajectory through the LGN. **c** Raw data (1 s segments) at each position along the trajectory of the central electrode in Patient #1. The depth relates to a preoperatively defined anatomical target. In Patient #1 spiking activity was apparent from −2mm (indicated by the dashed red line) to +4.5 mm. **d** Power-spectrum at one depth while the patient viewed an 8 Hz flickering light source. There were clear 8 Hz tracking and several harmonics. **e** The ratio of power 8 Hz to 5 Hz of the two microelectrodes (C1, central and P1, posterior) in Patient #1. Visually driven activity occurred from −2.0 to +3.5 mm, and was used to estimate the upper and lower boundaries of the LGN. **f** At each depth/electrode we sorted spike waveforms using WaveClus3. The auto-correlograms, cross-correlograms (black), and the waveforms are shown from the three units at a depth of +1.5 mm (1 single unit, 2 multi-units, see Methods). Error bars indicate ±1 standard deviation. In Patient #1, we recorded from 3 single-units and 13 multi-units. **g** A zoomed in view of the spiking responses from microelectrode P1 in response to 8 Hz flicker. A single-unit (red spikes) showed bursts of spiking whereas a simultaneously recorded multi-unit (blue spikes) fired predominantly to the opposite phase of the flicker. In this subject, the synchronization between the stimulus and the recording system was not present and we therefore estimated the stimulus phase (indicated by the black and gray horizontal bars above the graph). The auto-correlation function (**h**) and power spectrum (**i**) of the single-unit shown in (**g**). Bursting activity caused a peak at approximately 170 Hz. The bursts in Patient #1 had a typical inter-spike interval of 4.0–6.0 ms, slightly longer than in previous studies (e.g., ref. ^32^). Error-bars indicate ±1 s.e.m. Source data are provided as a Source Data file.

Two patients were unilaterally implanted with a DBS electrode in the LGN under local anesthesia (Fig.1a) as treatment for epilepsy originating in the occipital lobe^16^. Both patients had normal vision as assessed by pre- and postoperative perimetry (Fig. S1). Prior to implantation of the DBS electrode, two high-impedance microelectrodes (Fig.1b) were used to monitor spiking activity to determine the upper and lower boundaries of the LGN. We recorded activity at several depths along the trajectory of the microelectrodes (Fig.1c).

### Responses to flickering light in Patient #1

In Patient #1 the LGN activity was mapped with a handheld light source flickering at 8 Hz. Clear 8 Hz tracking was present in the power spectra of signals from both microelectrodes (Fig.1d, e). The distance between the top and bottom boundaries was 5.0 mm, which agrees well with estimates based on MRI^17,18^. At each depth/electrode we classified spikes as single- or multi-units by examining the inter-spike interval distribution, the auto-correlogram, and the waveform shape and consistency (Fig. 1f). Figure 1g exemplifies the spiking responses of an LGN single unit to the flickering light stimulus. The cell responded to one phase of the light-flicker with a repeatable burst of spikes with a firing rate of approximately 180 Hz, which was visible in the autocorrelation function (Fig.1g, h). The light stimulus generally elicited bursting responses of all single units in this patient, with frequencies of 150–230 Hz (Fig. S2A). Previous studies reported gamma oscillations in the LGN of anesthetized cats (e.g., refs. ^19,20^) but we did not observe strong gamma oscillations in either patient (Fig. S2B–E).

### Receptive-field mapping in Patient #2

**Fig. 2:**
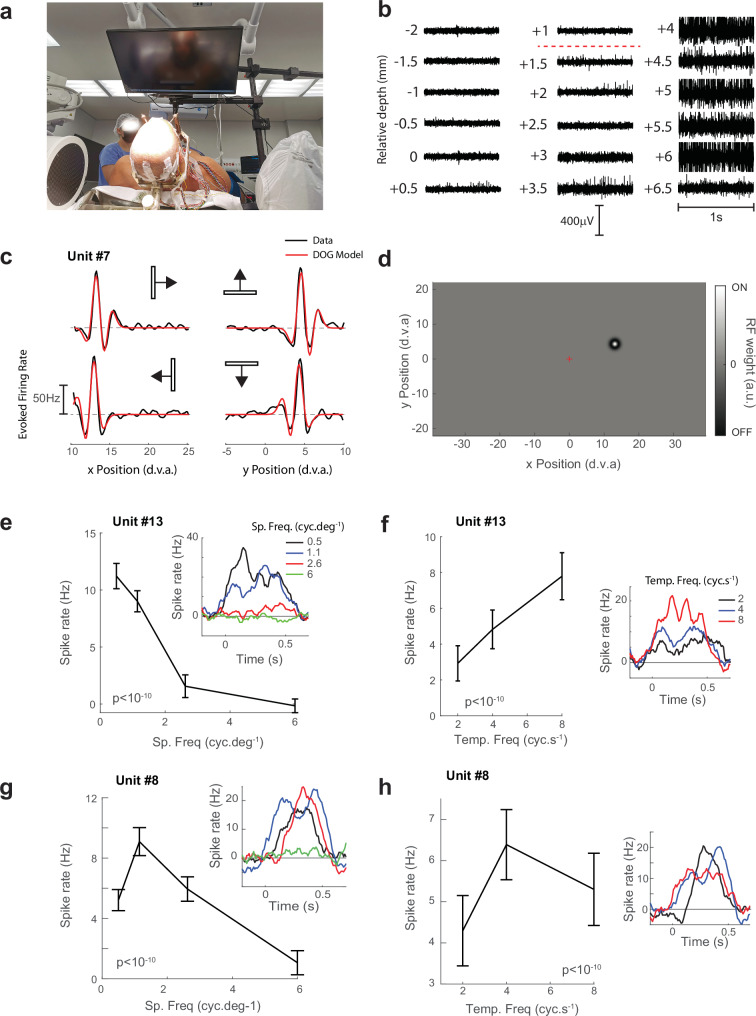
Tuning properties of LGN neurons in Patient #2. **a** The experimental set-up for Patient #2. The patient was supine and viewed a LCD monitor at a distance of 36.5 cm. **b** Activity from electrode C2. Spiking could be observed starting at +1.5 mm relative to the MRI-based anatomical target. We ran experimental sessions at depths of +4, +4.5 and +5.5 mm. **c** Black traces represent the average responses of Unit #7 to four different directions of the RF mapping luminance bar. Note that the x-axis refers to the spatial position of the light bar. The red trace shows the best-fitting DOG model (r^2^ = 0.91). **d** The RF model that provided the best fit to the data in (**c**). The gray rectangle illustrates the size of the CRT monitor. The patient fixated in the center (position (0,0), d.v.a. = degrees of visual angle). The RF was ON centered and located in the upper-right quadrant at an eccentricity of 13.9°. The size of the ON center was 1.3 d.v.a (Full-Width at Half Maximum (FWHM) of the fitted Gaussian, see Methods) and the OFF surround was 2.1 d.v.a. FWHM. **e** Responses from Unit #13 to drifting grating stimuli across spatial frequencies (*n* = 24 trials per frequency). Error-bars represent 1 s.e.m. in all panels. The mean activity differed significantly between spatial frequencies, pooled across temporal frequencies and directions (the p-value comes from a Poisson ANOVA). The inset shows the response elicited by different spatial frequencies. **f** Responses from the same unit grouped by temporal frequency. (*n* = 32 trials per frequency). **g** Responses from Unit #8, which preferred intermediate spatial frequencies (*n* = 24). **h** Temporal frequency responses of Unit #8 (*n* = 32). Source data are provided as a Source Data file.

In addition to the flickering light source (Supplemental Video 1), we presented visual stimuli to Patient #2 on a calibrated LCD monitor (Fig.2a). In this patient, spiking was apparent from +0.5 mm to +6.5 mm caudal to the magnetic resonance imaging (MRI)-based anatomical target (Fig.2b). We presented several visual stimuli at three electrode depths (+4.0 mm, +4.5 mm and +5.5 mm). While the subject directed his gaze to a fixation point, we mapped receptive fields using drifting bars (Fig.2c, Fig. S3) and modeled them as a difference-of-Gaussians (Fig. S3d). Figure 2c shows example fits of the model to the responses from Unit #7. The model provided an excellent fit to the responses (r^2^ = 0.91). The unit had an ON-centered RF at an eccentricity of 13.9 degrees of visual angle (d.v.a) (Fig. 2d) with a central sub-unit size of 0.57 d.v.a (standard deviation). The model also estimated the latency of the visual response of 44 ms (see Methods). In total, we obtained good quality fits to the RFs of four units (r^2^ greater than 0.5) (Fig. S4).

### Temporal and spatial-frequency tuning in human LGN

We examined the tuning of the neurons with drifting sine-wave gratings, focusing on spatial- and temporal-frequency tuning. An example unit (#13), illustrated in Fig. 2e, f, responded most strongly to low spatial frequencies and high temporal frequencies. It tracked the black-white phases of the grating with a temporal frequency of 8 cyc.s^−1^ and preferred the ipsilateral eye. These findings suggest that it was recorded in magnocellular layer 2 (see Fig. S5 for measurements of other neurons). Unit #8 on the other hand preferred intermediate spatial and temporal frequencies (Fig. 2g, h) suggesting that it may be located in a parvocellular layer. Similar responses were observed for other units (e.g., Unit #9, #11, Fig. S5). Responses at the highest spatial frequency that we tested (6 cyc.deg^-1^) were weak in all cells (Fig. S5). This was likely due to the larger eccentricities of the RFs and the properties of the display in the operation room, which reduced the grating contrast at this higher spatial frequency. We list the tuning of all LGN neurons to spatial and temporal frequency in Table S1 and provide putative layer assignments.

### Red-green opponent cells in human LGN

**Fig. 3:**
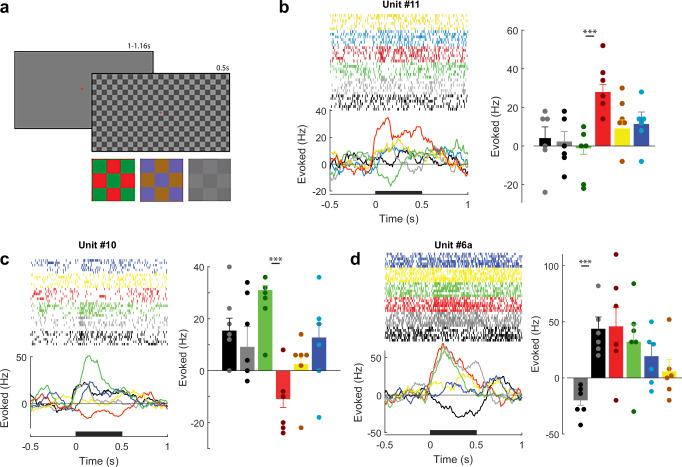
Red-Green opponent responses in human LGN. **a** The checkerboard stimuli used to assess color tuning and ocularity. Red-green, blue-yellow, and high and low-contrast achromatic checkerboards were shown binocularly. **b** Time course, raster-plots and mean activity of Unit #11 elicited by the checkerboards in a time-window from 0–500 ms (black bar). The units gave opposite responses to the red and green phases of the checkerboard. The colors of the bars and traces represent the color of the check in the RF. Gray bars represent the activity evoked by the white checks of the high-contrast achromatic grating (responses to the low contrast checkerboards are not shown). Dots indicate individual trials (*n* = 6). Error-bars represent 1 s.e.m. Asterisks indicate significant differences between responses to opposite-phases of each grating (***: t-test, two-sided, Bonferroni correction applied, *p* < 0.001). **c** Responses of Unit #10, which also showed red-green opponency. **d** Responses from unit #6a, which gave opposite responses to the black and white phases of the checkerboard. Source data are provided as a Source Data file.

To examine color tuning, we presented reversing checkerboard stimuli of different color contrasts (black-white, red-green, and yellow-blue, Fig.3a). Units #10 and #11 exhibited red-green opponency. Unit #11 was excited when the check in the RF was red and inhibited when it was green and Unit #10 had the opposite color preference (Fig.3b, c). Hence, red-green opponent cells exist in the human LGN and it seems likely that these units were in the parvocellular layers of the LGN, where red-green opponency is prominent in monkeys. Multi-unit recordings at some of the electrode sites responded oppositely to the two phases of black-white checkerboards (e.g., Unit #6a, Fig.3d). Both phases of the red-green checkerboard elicited excitation, but it is conceivable that neurons that were tuned to opposite phases of the checkerboard contributed to the multi-unit activity. The responses elicited by the checkerboard stimuli of all units are shown in Fig. S6. Most cells responded only weakly to the yellow/blue checkboards, and we did not observe units with different responses to the two phases of this stimulus. This finding is in accordance with the small proportion of blue-ON/OFF neurons in the LGN of primates, which are mainly found in the koniocellular layers^21,22^.

### The LGN responses to monocular stimulation and the effects of eye closure

**Fig. 4:**
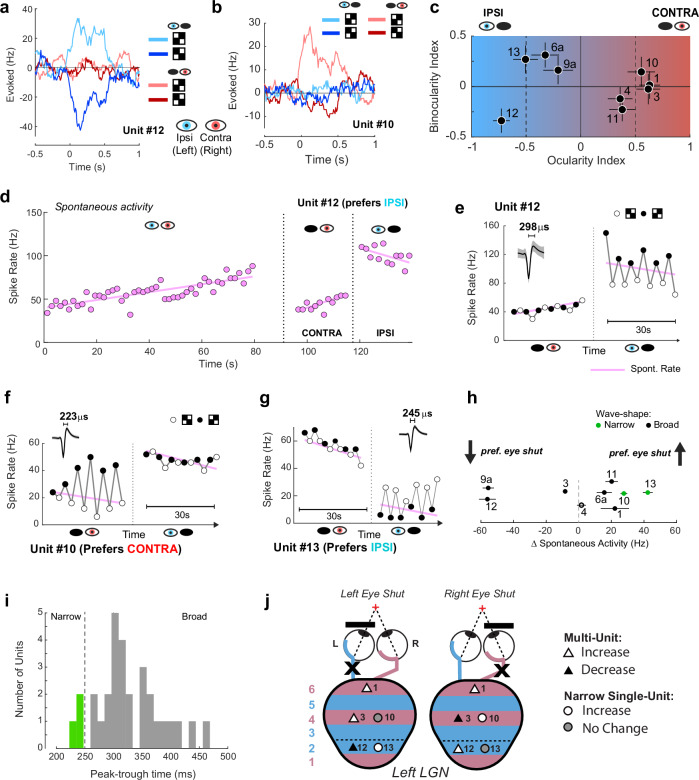
LGN activity is modulated by eye closure. **a** Responses of Unit #12 to monocularly presented checkerboards. **b** Responses from the simultaneously recorded Unit #10. **c** Ocularity and Binocularity index values from units that were tested with monocular stimuli. Error-bars indicate of ±1 bootstrap estimate of s.e.m. **d** Spontaneous activity of ipsilateral preferring unit #12. Each dot shows a trial. The pink line shows the best fitting linear regression. Variations in spike isolation were not responsible for the drift in spontaneous rate (Fig. S7a). **e** Response of Unit #12 to monocular stimuli. Black and white dots indicate responses to opposite phases of the checkerboard. The pink line is the regression line from (**d**). The inset shows the mean waveform and standard deviation (shaded region in all panels). **f** Responses from unit #10 with a relatively narrow waveform. Spontaneous activity increased when the preferred eye was shut. **g** Unit #13 had a narrow waveform and increased baseline activity when the preferred eye was shut. This unit was recorded simultaneously with units in (**e**, **f**). **h** The effects of eye-closure on spontaneous activity across the population. ΔSpontaneous activity was calculated as the firing rate with the preferred eye open minus the non-preferred eye open. The two units with the narrowest waveforms (peak-trough <250 µS, Units #10, #13) strongly decreased their activity when the preferred eye was shut. Vertical offsets were added to improve visibility. Error bars indicate ±1 s.e.m. **i** Peak-to-trough distances from all units in both patients. The sample was too small for a test of bimodality of the distribution. **j** Summary of effects of eye closure on spontaneous activity in the left LGN. Only strongly monocular units are shown (|OCIX | > 0.5). Cells were classified as having increased/decreased activity if the absolute change in spontaneous firing rate was greater than 5 Hz or larger than 25%. The two cells with the narrowest waveforms (circles) increased activity when the preferred eye was shut. The other units decreased (increased) their firing rate when their preferred eye was shut (open). Unit #1 was exceptional because the spontaneous firing rate increased in both monocular conditions. Source data are provided as a Source Data file.

We examined the responses to monocularly-presented black-white checkerboards by asking the patient (via written instruction on the screen) to shut one eye. The selectivity for the two eyes differed between cells. For example, unit #12 only responded to stimulation in the ipsilateral (left) eye, with excitatory responses to one phase of the black-white checkerboard and inhibitory responses to the opposite phase (Fig.4a) (spike-isolation remained consistent during monocular and binocular presentation, Fig. S7a). We quantified eye dominance with the ocularity index (OCIX, See Methods), which is positive for neurons driven by the contralateral eye and negative for neurons driven by the ipsilateral eye. Unit #12 had an OCIX of −0.73, indicating a strong ipsilateral response. Other cells were driven by the contralateral eye, such as Unit #10 with an OCIX of 0.56 (Fig.4b). Most units were predominantly driven by stimulation of one eye (|OCIX | > 0.5, Fig.4c), but some multi-unit recording sites also responded to the non-dominant eye (e.g., Units #4, 6a, 9, 11). It is possible that single cells with opposite eye-preferences contributed to these multi-units.

We also examined the interaction between the dominant and non-dominant eyes. We computed a binocularity index (BINOIX), which was negative if the binocular response was less than the dominant monocular response (binocular suppression) and positive if the opposite was true (binocular facilitation). Most cells had BINOIX values close to zero (Fig. 4c), Unit #12 was the only unit with significant binocular suppression (t-test, *p* = 0.02) and unit #6a was the only one with significant facilitation (t-test, *p* = 0.04). We used the eye-preferences in combination with the tuning preferences to putatively assign units to layers (Table S1). Unit #12, for example, was most likely in layer 2, where neurons prefer higher speeds and ipsilateral eye stimulation.

To our surprise, the spontaneous activity of many LGN cells depended strongly on the closure of one eye. An example is Unit #12, which preferred ipsilateral eye stimulation (Fig.4a, d). Shutting the dominant, ipsilateral eye decreased the spontaneous firing rate (from 58 to 45 Hz, t-test, *p* < 0.001) and shutting the non-dominant eye caused the activity to increase to 100 Hz (compared to both eyes open; t-test, *p* < 0.001). Responses to monocular stimulation of the preferred eye built upon the increased baseline activity, leading to a much higher firing rate upon dominant eye stimulation compared to non-dominant eye stimulation (Fig. 4e). We observed several units that increased their baseline activity when the dominant eye was shut. One example is Unit #10 (Fig.4f), which had a narrower waveform than most other units (Table S1, Fig. S7b). In the cortex, narrow waveforms belong to interneurons^23,24^, but this relationship has, to our knowledge, not yet been established for the thalamus. Nevertheless, when we examined the other unit (#13) with a narrow waveform, we also observed increased activity if the preferred eye was shut (Fig.4g, Fig. S7b). Although our sample is too small for strong conclusions, it is remarkable that the two cells with the narrowest waveforms exhibited the strongest increases in spontaneous activity when the preferred eye was shut (Fig. 4h).

## Discussion

### Receptive-field characteristics of human LGN neurons

We here exploited a unique opportunity to record from neurons in the LGN of two awake human participants. Due to the time constraints of recording intra-operatively it was not possible to record from a large sample of neurons, but we were able to verify that several RF tuning properties of LGN neurons in monkeys generalize to humans. Notably, we confirmed that some human LGN neurons are well described by a difference-of-Gaussians model, and we confirmed the existence of red-green opponent cells in the parvocellular layers of human LGN. The RFs studied by us were larger than those recorded in non-human primates. These earlier studies mapped RF sizes by hand, or reported the radius of the central sub-unit from a difference-of-Gaussians (DoG) model fit to the responses to drifting gratings of different spatial frequencies^6^. We here estimated RF size using drifting bars, which is more time-efficient and also allows the fitting of a DoG model. The best-fitted RF of the present study had an eccentricity of 13.8 degrees and was likely parvocellular. The central sub-unit was 0.57 deg, which is larger than the values of 0.06–0.15 degrees that were measured at this eccentricity in anesthetized, paralyzed monkeys^6,25^. It is conceivable that we overestimated sizes of the RFs because the subject made small eye-movements. The LGN cells in our sample also preferred lower spatial frequencies than reported in macaques. The putative magnocellular neurons showed low-pass characteristics and the parvocellular neurons preferred spatial frequencies in the range 1–3 cyc/deg, which is lower than the more typical range in primates of 3–10 cyc/deg^6^. We note, however, that the eccentricity of the RFs in our study was larger than 10 degrees, whereas studies in monkeys mostly focused on the parafoveal region. Furthermore, our LCD had a resolution of ~24 pixels per degree so that an aliasing effect decreased the contrast of the drifting gratings with the highest spatial frequency of 6 cycles/degree.

### Layer assignments

We putatively assigned the neurons to the LGN layers by considering three sources of information: (1) the position of the electrode along the dorsal-ventral axis (2) the ocularity of the response of units and that of other units recorded at the same electrode depth and (3) the RF tuning properties of cells and other neurons at the same depth. For example, units #12 and #13 preferred high speeds and were driven by the ipsilateral eye. We confidently assigned them to layer 2, which is the only layer containing cells with that combination of response properties in monkeys. Nevertheless, it was not always easy to unambiguously assign units to a layer. In some cases, we estimated the layer based on the distance to other cells. For example, our measurements did not allow us to assign unit #5 unambiguously to a layer, but we could assign more dorsal and more ventral units to layers 6 and 4, respectively. Furthermore, a multi-unit recorded at the same depth as unit #5 responded most strongly to the ipsilateral eye, increasing our confidence that unit #5 was also in layer 5. Some of these layer assignments should therefore be viewed as best estimates rather than definitive.

### Binocular interactions in the human LGN

The LGN is a predominantly monocular structure and in our dataset all single-units, and some multi-units, were predominantly driven by one eye. We did however observe an effect of stimulation of the non-preferred eye on the binocular response in two multi-units. Binocular stimulation decreased the activity of unit #12 by approximately 50%. Binocular suppression has been observed in macaque LGN^9,26^ but is less common than in the cat^10,27,28^. For example, Xue et al.^28^. reported that 75% of cat LGN neurons show binocular suppression. In primates, only 25% of isolated cells show suppression^9^ although the percentage is higher for multi-unit activity^26^. These suppressive influences could be mediated by interneurons within the LGN or projections from the thalamic reticular nucleus or the primary visual cortex. Results from psychophysical studies in humans have provided evidence for these suppressive interactions between the images conveyed by the two eyes at a monocular level of processing^29,30^. We also observed one multi-unit which showed binocular facilitation. However, we hesitate to draw strong conclusions from this observation, because we could not exclude the possibility that we recorded from a mixture of neurons driven by the two eyes.

### Spontaneous activity changes during voluntary eye closure

We observed a strong influence of voluntary eye closure on the spontaneous activity levels in the human LGN. The spontaneous activity of some cells decreased when the preferred eye was shut and increased when the non-preferred eye was shut. The reduction in activity upon closure of the preferred eye could be due to a reduction in the retinal input because the patient saw a gray screen when the eye was open. However, the increase in spontaneous activity upon closure of the non-preferred eye suggests a suppressive influence of this eye. We also observed the opposite effect in some of the units, which increased their spontaneous activity when the preferred eye was closed. This effect was particularly pronounced for the two cells with the narrowest waveforms (Fig. 4i). In the cortex interneurons have the narrowest waveforms, raising the intriguing possibility that these cells were interneurons. The shortest peak-to-trough times in our sample (223 and 245 µs) were somewhat longer than the values of cortical interneurons^24^ (150–200 µs), but little is known about action potential waveforms in the human thalamus. Under the assumption that the two cells with the narrowest waveforms were interneurons, our results suggest an interocular gain control mechanism in which local interneurons suppress the activity of cells that are normally driven by the closed eye (Fig. 4j). We do not know to which degree the change in spontaneous LGN firing rates reflects the altered input from the eye^29,30^ and/or top-down signals from brain regions related to voluntary eye closure, because the effects of voluntary eye closure on neuronal activity have not been tested in animal models. Nevertheless, the large changes in spontaneous LGN activity caused by the closure of one eye are remarkable, because visual perception is hardly altered despite a halving of the input to the visual system. We look forward to future studies using similar techniques, to provide additional insight into the early visual pathways of the human brain.

## Methods

### Patient details

Patient #1 has been described previously^16^. Briefly, she was a 60-year-old female patient presenting with generalized tonic-clonic seizures at age 8, which persisted until she turned 35. Since then, the characteristics of her seizures changed completely. Seizures started when she saw a white cloud in the left visual field that lasted 10–20 s (focal aware visual seizure, FAVSs), frequently evolving to focal impaired awareness seizure (FIASs moaning, bilateral hand automatisms, rubbing her eyes), with manifestations typical of temporal lobe epilepsy. Ref. ^16^ describes the details of the pre-surgical investigation. Medication at the time of surgery was lamotrigine 200 mg twice per day, levetiracetam 1125 mg twice per day, and clobazam 20 mg once per day.

Patient #2 was male, 45 years old and he experienced his first seizures at an age of 7 years. He was diagnosed with a left occipital lobe tumor at age 14. The tumor was completely resected and was diagnosed as a low-grade glioma. He presented with seizures occurring at a mean frequency of 5 mild seizures per day and 2 strong seizures per week. Seizures were typically associated with bright phosphenes in the right visual field, evolving with disperceptive manifestations, including automatisms of the right hand. Video-EEG suggested epileptogenic activity starting in the left occipital lobe. PET-CT revealed hypometabolism in the left occipital lobe and MRI images showed an area of encephalomalacia/gliosis in the left occipital lobe (secondary to the previous tumor and its surgical resection). Perimetry revealed a pre-operative linear nasal scotoma in the left eye, though this was not replicated in the post-surgical perimetry (Fig. S1). Neuropsychological evaluation was suggestive of bilateral frontotemporal dysfunction. SF-36 (scale for quality of life): impairment in all domains except physical and emotional limitations and pain. GAF (global assessment of functioning): 70-61. Medications at time of surgery: levetiracetam 500 mg BID, lamotrigine 100 mg BID, clobazam 10 mg SID. Given the inherent risk of hemianopia following resective surgery in the occipital lobe, the proposed treatment was to implant the left LGN with a DBS electrode for low-amplitude stimulation to prevent epileptogenic activity formation in the left occipital lobe. The study was approved by the Brazilian National Research Ethics Committee (CONEP), report #862348. The study was also registered in the Brazilian Registry of Clinical Trials (ReBEC) and written permission was obtained from both patients.

### Trajectory planning

We determined the target coordinates based on stereotactic computed tomography (CT) and frameless MR (proton density, T2-weighted, and T1-weighted axial slices) merged images, relying mainly on direct visualization of the LGN, but also on the tractography. This structure was identified on an axial slice showing the superior colliculi and the subthalamic and red nuclei, as an area of high signal intensity relative to that of the surrounding white matter, i.e., the posterior limb of the internal capsule, anteromedially, and the optic radiations, laterally (proton density and T2-weighted axial slices). Considering the triangular shape of this nucleus, with a flat base and the apex pointed superolaterally, the trajectory was planned to traverse its major vertical axis, which is about 5.0 mm long, as seen on coronal images.

Physiological mapping for target confirmation was performed with the patient awake, under local anesthesia. Two microelectrode trajectories, 2.0 mm apart, were performed. One centered on the anatomical target as defined by the pre-surgical MRI scan (Electrodes C1 and C2) and one situated posterior to the target by 2 mm in Patient #1 (Electrode P1) and one situated anterolateral to this coordinate by 2 mm in Patient #2 (Electrode A2). Each probe comprised a high-impedance sharp electrode, used to measure spiking, and a larger contact used primarily for electrical macrostimulation. The electrodes were stepped through the brain in 0.5 mm steps and recordings were made from 7.0 mm above (−) to 7.0 mm below (+) the target. The responses to photic stimulation (8 Hz light flashes, square-wave), as well as those from macrostimulation, were used to determine the superior and inferior borders of the LGN. This determination was made by the clinical team. Macrostimulation (150 Hz, 1.0 ms pulse-width, train duration of 2 sec, amplitude <0.2 mA), induced phosphenes in the left visual field in Patient #1 and the right visual field in Patient #2 and photic stimulation induced bursting activity was observed from −2mm to +3.5 mm (Patient #1) and +1.5 mm to + 5.5 mm (Patient #2), defining the LGN borders. In Patient #2, high-frequency stimulation at the target induced a focal aware seizure evolving to focal impaired awareness seizure, which was recorded on the scalp EEG over the left occipital region. The contact 1 of the DBS lead (Sensight model, Medtronic) was placed at the center of the physiological target (+3.5 mm below the anatomical target). After general anesthesia, the lead was connected to the pulse generator (Percept, Medtronic), placed in the right infraclavicular region, through the subcutaneous extension cable.

### Data acquisition

Patient #1: Electrophysiological data was obtained from two MER electrodes (Medtronic) and amplified with the LeadPoint system (Medtronic). Analog filters were set to 500 Hz (high-pass) and 5000 Hz (low-pass). The data was then sampled at 24 Khz.

Patient #2: Electrophysiological data from the two InoMed MicroMacro electrodes (impedance of the micro-tips was in the range 0.8–0.9 MΩ) were amplified using an ISIS MER system (InoMed), analog filters were set to 120 Hz (high-pass) and 5000 Hz (low-pass). The data were sampled at 20 KHz/16bits and stored for offline analysis. The electrophysiological data was synchronized to the visual presentation of the stimuli using a photodiode (Vishay, BPW21R). The signal from the diode was amplified using a custom-built amplifier (Joris Koppens, NIN). The amplifier box contained a circuit for detecting decrements in light which triggered a 5 V TTL pulse. These pulses were sent to the digital input of the InoMed amplifier and were sampled using the same clock as the electrophysiological data. The beginnings of individual recording sessions were marked with a signal by flickering the screen at the diode location with a pre-designated pattern. The onset of each visual stimulus was accompanied by a luminance decrement of the screen at the location of the diode (invisible to the patient as this region was shielded with tape, see Fig. 2a).

### Spike-sorting

We sorted spikes separately for each experimental session using WaveClus 3^31^. The raw data from each high-impedance electrode were high-pass filtered above 300 Hz. Threshold crossings were detected using a threshold of 4.5 times the unbiased estimate of the median absolute deviation of the signal. The threshold-crossing detection algorithm had a refractory period of 1 ms. Spike waveforms were stored as 35 samples (15 pre-threshold, 20 post-threshold) with a total duration of 1.5 ms (Patient #1) or 1.8 ms (Patient #2). Each waveform was up-sampled 5 times using cubic-spline interpolation before the dimensionality of the entire set of waveforms was reduced by using a wavelet decomposition. Wavelet features were clustered using super-paramagnetic clustering and clusters were visually inspected. Clusters with very similar waveforms were merged. Wave-forms were examined between experimental-sessions from the same electrode and cells were identified that were measured across sessions. Units were classified as either multi-unit or single-unit on the basis of the inter-spike interval distribution and the waveform. Single-units were defined as units with less than 3% of spikes having an inter-spike interval of less than 3 ms and with a signal-to-noise ratio (defined as the maximum absolute amplitude of the mean spike waveform divided by the mean standard deviation of the waveforms) greater than 5. We defined cells as having a narrow waveform if the peak-to-trough time of the average waveform was <250 μs. Narrow waveform cells have been shown to be more likely to be fast-spiking interneurons in the hippocampus and cortex^23,24^. Little is known about the association between cell-classes and waveform in the LGN.

### Receptive field mapping

We mapped RFs using a drifting luminance defined bar. On each trial the white bar (15 d.v.a in length, 1 d.v.a. in width, 60 cd.m^−2^) drifted in one of the 4 cardinal directions for 1 s at a speed of 15 d.v.a. per second. The bar-sweep was followed by an inter-trial interval consisting of only the gray background (luminance = 30.5 cd.m^−2^) and fixation cross for 0.35–0.48 s (uniform distribution, randomly selected on each trial). In each session there were 10 repeats of each direction. The bar sweep was centered on the best guess of the receptive field location estimated during manual mapping, the patient was instructed to fixate on the central cross throughout. Each session lasted approximately 56 s.

Spiking data from each unit were analyzed by binning spike-times from each trial into 10 ms bins and subtracting the spontaneous rate, measured in a time-window from −0.2s–0s relative to the onset of the bar. The resulting peri-stimulus time histogram (PSTH) was corrected for possible changes in the fixation position of the patient by cross-correlation. If the patient fixated at slightly different positions on each trial, then this would result in a shift of the PSTH as the bar would arrive in the RF at a different time-point. We corrected for these unknown shifts by shifting the PSTH on each trial in time so that it maximally correlated with the average trace across trials. For each direction of the drifting bar, we calculated the average PSTH across the 10 repeats and smoothed this trace with a 100 ms moving average window. We then calculated the lag (in the range 200 to +200 ms, which equates to ±3 d.v.a fixation offset) that produced the maximum correlation between the PSTH on each trial (smoothed with the same window) and the average PSTH. The best fitting lags had a standard deviation of 0.44 d.v.a. in the x-direction and 0.21 d.v.a. in the y-direction, suggesting that the subject’s fixation on the fixation point was relatively accurate. The PSTH of each trial was then shifted by the best fitting lag to co-register each trial’s PSTH with the average trace. After co-registration we took the mean across repeats to generate a mean PSTH vector: ${v}_{{{\mathrm{dir}}}}$ for each of four directions dir. Mean PSTHs were converted from a time base to a spatial base so that responses could be expressed as a function of the spatial position *sp* of the bar. We fit these response profiles as a function of space with a difference-of-Gaussians model, which has been used previously to model LGN receptive fields in the monkey^5^. The two dimensional RF(*x*,*y*), where *x* and *y* are the Cartesian spatial coordinates of the RF center in d.v.a, was assumed to be circularly symmetrical. We furthermore assumed that the response was driven (i) by the luminance increments and decrements caused by the bar sweep, i.e., the spatial differential of the bar stimulus, *bar_diff*, (illustrated below the x-axis of Fig. S3d) and (ii) the stationary luminance profile. The dynamic component of the response $v({{\mathrm{sp}}},{{\mathrm{dir}}})$ was modeled as a convolution of a 1D projection of the RF, ${w}_{{{\mathrm{RF}}}}\left({{\mathrm{sp}}},{{\mathrm{dir}}}\right)$, with *bar_diff* over space *sp*: ${m}_{{{\mathrm{dir}}}}b({{\mathrm{sp}}})*w\left({{\mathrm{sp}}},{{\mathrm{dir}}}\right)$. The influence of the stationary position was modeled as another Gaussian, *G*_*Stat*_, resulting in

1 $$\begin{array}{l}v\left({sp},{dir}\right)={m}_{{dir}}{{bar}}\_{{diff}\left({sp}\right)} * w\left({sp},{dir}\right)+n{G}_{{Stat}}\left({\mu }_{t}+{sp},{\sigma }_{3}\right)\\ {w}_{{RF}}\left({sp},{dir}\right)={G}_{{Center}}\left({\mu }_{x,y}+s,{\sigma }_{1}\right)-k{G}_{{Surround}}\left({\mu }_{x,y}+s,{\sigma }_{2}\right)\\ {{bar}}\_{{diff}\left({sp}\right)}=\,\left[\cdots,\,0,\,0,\,0,\,1,\,0,\,0,\,0,\,\cdots,\,0,\,0,-1000,\,\cdots \right]\end{array}$$

Where *G*_Center_ *and G*_Surround_ are Gaussian functions with the same mean ${\mu }_{x,y}$, and ${\sigma }_{1}$ and ${\sigma }_{2}$ are standard deviations, and *k* is a scaling factor which controls the contribution of the surround. The variable *s* is the spatial offset of the RF center due to visual latency of LGN cells. The delay *s* caused by synaptic and conduction delays between the photo-receptors of the retina and LGN cells results in a RF shift along the trajectory of the bar, because the bar will have moved on when the LGN unit starts to respond. To account for this effect, the RF center was shifted by a term s backwards along the trajectory of the bar. The spatial shift can be converted to a temporal latency based on the speed of the bar to generate an estimate of the response latency of the unit. The time shift was constrained to lie in the physiological range of 30–60 ms which equates to shifts of 0.45 to 0.9 d.v.a, given the bar speed of 15 d.v.a./s. The variable *m*_dir_ is a scaling factor which captures the responsivity of the cell. The Gaussian that describes the stationary response *G*_Stat_ was also centered at ${\mu }_{x,y}$ with a standard deviation, ${\sigma }_{3}$, and an amplitude that was determined by the parameter $n$. We simultaneously fit the responses elicited by all four bar directions, using non-linear least squares fitting using the Nelder-Mead algorithm (fminsearchbnd.m in MATLAB 2021b). To model ON and OFF cells the vector *bar_diff* was used (ON cells) or inverted (OFF cells). We initialized the fits with various estimates of *x* and *y* parameters on a grid around the maximum of the response averaged over opposite directions of bar movement and chose the model with the highest r^2^.

### Determination of spatial and temporal frequency tuning with drifting gratings

We measured the tuning of the LGN cells to spatial and temporal frequency with drifting gratings. Each grating was shown for 0.5 s with a 0.3 s inter-trial interval. The grating stimuli drifted in one of 8 directions (0⁰, 45⁰, 90⁰, …, 315⁰) at a temporal frequency of 2, 4 or 8 cycles per second. The spatial frequency of the grating was 0.5, 1.1, 2.6 or 6 cycles per degree. The grating was shown through a circular aperture of 12⁰ diameter on a gray screen of the average luminance (30.5 cd.m^-2^) and had a contrast of 80%. There were 96 trials (one of each combination of direction, speed, and spatial frequency) presented in a pseudorandom order. The patient was instructed to fixate throughout the session on the central fixation cross. We ran two sessions at depths +4.5 mm and +5.5 mm. Due to a problem with the acquisition system, the data from the first 20 trials of the session at +5.5 mm were not recorded. For each trial we calculated the evoked activity as the mean spike-rate in a window from 0 to 0.5 s after stimulus onset, after subtraction of a linear estimate of the baseline activity (as in Fig. 4d). For the analysis of spatial frequency tuning, we averaged across temporal frequency and directions. Similarly, we averaged across spatial frequency and direction to analyze temporal frequency tuning. We do not report orientation or direction selectivity analyses here due to the low number of trials available for analysis at each direction/orientation.

### Color responses and monocular stimulation

We assessed the responses to different colors using three checkerboards: black-white, red-green and blue-yellow. Each check was 2 d.v.a. in size. The luminance of the red, green, yellow and blue colors was matched using a photometer (Minolta) to the background gray luminance (30.5 cd.m^−2^). This luminance could not be achieved using the blue-channel only (which had a maximum luminance of 13.1 cd.m^-2^) and we therefore added equal amounts of red/green channel to the blue-channel until the target luminance was reached. We did not have access to a spectrophotometer and the stimuli were therefore not cone-isolating. The contrast of the high contrast black-white checkerboard was 57%, and we also presented a version with 10% contrast. The phase of the checkerboard could be 0° or 180° (e.g., black-white or white-black). It was displayed for 0.5 s with an inter-trial interval consisting of the gray screen and fixation cross which lasted for 1 to 1.16 s. Each checkerboard was presented 6 times at each phase in a pseudorandom order, yielding a total of 48 presentations. Thereafter the patient was instructed by a text screen to shut the left eye. After visual verification of eye closure the patient saw 12 checkerboards (black-white, 57% contrast, alternating 0° or 180°) with the right eye (*contralateral* presentation). Then we instructed him to shut the right eye and open the left eye and we presented a further 12 checkerboards (*ipsilateral* presentation). Statistics were performed using independent samples t-tests on the absolute mean evoked (i.e., baseline corrected by removing a linear estimate of the baseline response) spike-rate between 0 and 0.5 s, the Satterthwaite correction for unequal variance was applied to calculate corrected degrees of freedom. Monocular cells were defined as those having a significant difference in response to left and right eye stimulation (*p* < 0.05). The ocularity index (OCIX) was calculated using the absolute mean evoked spike-rates from the ipsi- (I) and contralateral (C) presentations as:

2 $${{\mathrm{OCIX}}}=\,\frac{(C-I)}{(C+I)}$$

We also quantified the strength of binocular facilitation/suppression using the binocularity index (BINOIX). We identified the eye that yielded the strongest monocular response (M) and then compared this response to the average response to high contrast, binocularly viewed, black-white gratings (B):

3 $${{\mathrm{BINOIX}}}=\frac{(B-M)}{B+M}$$

### Reporting summary

Further information on research design is available in the Nature Portfolio Reporting Summary linked to this article.

## Supplementary information

Supplementary Information

Reporting Summary

Description of supplementary

Transparent Peer Review file

Supplementary Movie 1

## Source data

Source Data

## Data Availability

The data generated in this study have been deposited in the Open Science Framework database under accession code 10.17605/OSF.IO/FTGHE via https://osf.io/ftghe/. Source data are provided with this paper.

## Code availability

The code generated in this study have been deposited in the Open Science Framework database under accession code 10.17605/OSF.IO/FTGHE via https://osf.io/ftghe/.

## References

[1] Luco, C., Hoppe, A., Schweitzer, M., Vicufia, X. & Fantin, A. Visual field defects in vascular lesions of the lateral geniculate body. *J. Neurol. Neurosurg. Psychiatry* **55**, 12–15 (1992). 1548490 10.1136/jnnp.55.1.12 PMC488924

[2] Hubel, D. H. & Wiesel, T. N. Effects of varying stimulus size and color on single lateral geniculate cells in Rhesus monkeys. *Proc. Natl. Acad. Sci. USA* **55**, 1345–1346 (1966). 4960305 10.1073/pnas.55.6.1345 PMC224323

[3] Wiesel, T. N. & Hubel, D. H. Spatial and chromatic interactions in the lateral geniculate body of the rhesus monkey. *J. Neurophysiol.* **29**, 1115–1156 (1966). 4961644 10.1152/jn.1966.29.6.1115

[4] Hubel, D. H. & Wiesel, T. N. Integrative action in the cat’s lateral geniculate body. *J. Physiol.* **155**, 385–398.1 (1961). 13716436 10.1113/jphysiol.1961.sp006635 PMC1359861

[5] Jeffries, A. M., Killian, N. J. & Pezaris, J. S. Mapping the primate lateral geniculate nucleus: a review of experiments and methods. *J. Physiol. Paris* **108**, 3–10 (2014). 24270042 10.1016/j.jphysparis.2013.10.001 PMC5446894

[6] Derrington, A. M. & Lennie, P. Spatial and temporal contrast sensitivities of neurones in lateral geniculate nucleus of macaque. *J. Physiol.* **357**, 219–240 (1984). 6512690 10.1113/jphysiol.1984.sp015498 PMC1193256

[7] Hendry, S. H. & Reid, R. C. The koniocellular pathway in primate vision. *Annu. Rev. Neurosci.* **23**, 127–153 (2000). 10845061 10.1146/annurev.neuro.23.1.127

[8] Kaas, J. H., Guillery, R. W. & Allman, J. M. Some principles of organization in the dorsal lateral geniculate nucleus. *Brain. Behav. Evol.* **6**, 253–299 (1972). 4196831 10.1159/000123713

[9] Dougherty, K. et al. Binocular suppression in the macaque lateral geniculate nucleus reveals early competitive interactions between the eyes. *eNeuro* **8**, ENEURO.0364–20.2020 (2021). 33495241 10.1523/ENEURO.0364-20.2020 PMC8035044

[10] Sanderson, K. J., Bishop, P. O. & Darian-Smith, I. The properties of the binocular receptive fields of lateral geniculate neurons. *Exp. Brain Res.* **13**, 178–207 (1971). 4936711 10.1007/BF00234085

[11] Zeater, N., Cheong, S. K., Solomon, S. G., Dreher, B. & Martin, P. R. Binocular visual responses in the primate lateral geniculate nucleus. *Curr. Biol.* **25**, 3190–3195 (2015). 26778654 10.1016/j.cub.2015.10.033

[12] Belluccini, E., Zeater, N., Pietersen, A. N. J., Eiber, C. D. & Martin, P. R. Binocular summation in marmoset lateral geniculate nucleus. *Vis. Neurosci.* **36**, E012 (2019). 31718727 10.1017/S0952523819000099

[13] Tong, L., Guido, W., Tumosa, N., Spear, P. D. & Heidenreich, S. Binocular interactions in the cat’s dorsal lateral geniculate nucleus, II: effects on dominant-eye spatial-frequency and contrast processing. *Vis. Neurosci.* **8**, 557–566 (1992). 1586654 10.1017/s0952523800005654

[14] Haynes, J.-D., Deichmann, R. & Rees, G. Eye-specific effects of binocular rivalry in the human lateral geniculate nucleus. *Nature* **438**, 496–499 (2005). 16244649 10.1038/nature04169 PMC1351280

[15] Lehky, S. R. & Maunsell, J. H. No binocular rivalry in the LGN of alert macaque monkeys. *Vis. Res.* **36**, 1225–1234 (1996). 8711902 10.1016/0042-6989(95)00232-4

[16] Vilela-Filho, O., Silva-Filho, H. F., Goulart, L. C., Ragazzo, P. C. & Arruda, F. M. A new strategy for treating drug-resistant focal aware seizures: thalamic specific nuclei deep brain stimulation. Illustrative case. *J. Neurosurg. Case Lessons* **6**, CASE23303 (2023). 10.3171/CASE23303 PMC10555561 37728299

[17] Li, M. et al. Quantification of the human lateral geniculate nucleus in vivo using MR imaging based on morphometry: volume loss with age. *Am. J. Neuroradiol.* **33**, 915–921 (2012). 22245591 10.3174/ajnr.A2884 PMC7968799

[18] Horton, J. C., Landau, K., Maeder, P. & Hoyt, W. F. Magnetic resonance imaging of the human lateral geniculate body. *Arch. Neurol.* **47**, 1201–1206 (1990). 2241617 10.1001/archneur.1990.00530110059017

[19] Neuenschwander, S. & Singer, W. Long-range synchronization of oscillatory light responses in the cat retina and lateral geniculate nucleus. *Nature* **379**, 728–732 (1996). 8602219 10.1038/379728a0

[20] Neuenschwander, S. et al. On the functional role of gamma synchronization in the retinogeniculate system of the cat. *J. Neurosci.* **43**, 5204–5220 (2023). 37328291 10.1523/JNEUROSCI.1550-22.2023 PMC10342227

[21] White, A. J., Wilder, H. D., Goodchild, A. K., Sefton, A. J. & Martin, P. R. Segregation of receptive field properties in the lateral geniculate nucleus of a New-World monkey, the marmoset Callithrix jacchus. *J. Neurophysiol.* **80**, 2063–2076 (1998). 9772261 10.1152/jn.1998.80.4.2063

[22] Szmajda, B. A., Buzás, P., Fitzgibbon, T. & Martin, P. R. Geniculocortical relay of blue-off signals in the primate visual system. *Proc. Natl. Acad. Sci. USA* **103**, 19512–19517 (2006). 17158219 10.1073/pnas.0606970103 PMC1748257

[23] Henze, D. A. et al. Intracellular features predicted by extracellular recordings in the hippocampus in vivo. *J. Neurophysiol.* **84**, 390–400 (2000). 10899213 10.1152/jn.2000.84.1.390

[24] Mitchell, J. F., Sundberg, K. A. & Reynolds, J. H. Differential attention-dependent response modulation across cell classes in macaque visual area V4. *Neuron* **55**, 131–141 (2007). 17610822 10.1016/j.neuron.2007.06.018

[25] Kremers, J. & Weiss, S. Receptive field dimensions of lateral geniculate cells in the common marmoset (Callithrix jacchus). *Vis. Res.* **37**, 2171–2181 (1997). 9578900 10.1016/s0042-6989(97)00041-2

[26] Schroeder, C. E., Tenke, C. E., Arezzo, J. C. & Vaughan, H. G. Binocularity in the lateral geniculate nucleus of the alert macaque. *Brain Res.* **521**, 303–310 (1990). 2207668 10.1016/0006-8993(90)91556-v

[27] Sengpiel, F., Blakemore, C. & Harrad, R. Interocular suppression in the primary visual cortex: apossible neural basis of binocular rivalry. *Vis. Res.* **35**, 179–195 (1995). 7839615 10.1016/0042-6989(94)00125-6

[28] Xue, J. T., Ramoa, A. S., Carney, T. & Freeman, R. D. Binocular interaction in the dorsal lateral geniculate nucleus of the cat. *Exp. Brain Res.* **68**, 305–310 (1987). 3691703 10.1007/BF00248796

[29] Ding, J. & Sperling, G. A gain-control theory of binocular combination. *Proc. Natl. Acad. Sci. USA*. **103**, 1141–1146 (2006). 10.1073/pnas.0509629103 PMC1347993 16410354

[30] Baker, D. H., Meese, T. S. & Summers, R. J. Psychophysical evidence for two routes to suppression before binocular summation of signals in human vision. *Neuroscience* **146**, 435–448 (2007). 17346895 10.1016/j.neuroscience.2007.01.030

[31] Chaure, F. J., Rey, H. G. & Quian Quiroga, R. A novel and fully automatic spike-sorting implementation with variable number of features. *J. Neurophysiol.* **120**, 1859–1871 (2018). 29995603 10.1152/jn.00339.2018 PMC6230803

[32] Sanchez, A. N., Alitto, H. J., Rathbun, D. L., Fisher, T. G. & Usrey, W. M. Stimulus contrast modulates burst activity in the lateral geniculate nucleus. *Curr. Res. Neurobiol.* **4**, 100096 (2023). 37397805 10.1016/j.crneur.2023.100096 PMC10313900

