# Supplementary material for: Effects of eye closure on the spiking activity of human lateral geniculate neurons
Source: Nat Commun. 2025 Nov 24;16:10402. doi: 10.1038/s41467-025-65383-x (PMC12645034; doi:10.1038/s41467-025-65383-x)
Supplement: Supplementary file 1 — Supplementary Information [file 41467_2025_65383_MOESM1_ESM.pdf]

Supplementary Information

ID	Depth	Elec	RF	SUA/MUA	pk/trgh	SF	TF	Color	Eye	Layer
1	4	C2	~	MUA	293	~	~	Red ON #	CONTRA	6
2	4	C2	~	SUA	402	~	~	On/Off ***	Unknown	6
3	4	A2	~	MUA	381	~	~	Green/Red *	CONTRA	4/I
4	4	A2	~	MUA	468	~	~	Green/Red **	CONTRA	4/I
5	4.5	C2	<0	SUA	243	Unclear	Fast	~	~	5?
6a	4.5	C2	~	MUA	302	~	~	On/OFF ***	Mildly IPSI	5?
6b	4.5	C2	0.17	MUA	290	Unclear	Inter	~	~	5?
7	4.5	A2	0.91	SUA	320	LP	Fast	~	~	3
8	4.5	A2	0.56	SUA	282	Inter	Inter	~	~	3
9a	4.5	A2	~	MUA	305	~	~	Green ON #	IPSI	3
9b	4.5	A2	0.17	MUA	418	Inter	Inter	~	~	3
10	5.5	C2	0.29	SUA	223	LP/Inter	Unclear	Green/Red ***	CONTRA	4
11	5.5	C2	0.17	MUA	324	LP/Inter	Inter	Red/Green ***	CONTRA	4
12	5.5	A2	0.73	MUA	298	LP	Fast	No Tuning	IPSI	2
13	5.5	A2	0.62	SUA	245	LP	Fast	On/Off ***	IPSI	2

ID	Depth	Elec	RF	SUA/MUA	pk/trgh	SF	Speed	Color	Eye	Layer?
1	4	C2	~	MUA	293	~	~	Red ON #	CONTRA	6
2	4	C2	~	SUA	402	~	~	On/Off ***	Unknown	6
5	4.5	C2	<0	SUA	243	Unclear	Fast	~	~	5?
6a	4.5	C2	~	MUA	302	~	~	On/Off ***	IPSI	5?
6b	4.5	C2	0.17	MUA	290	Unclear	Inter	~	~	5?
10	5.5	C2	0.29	SUA	223	LP/Inter	Unclear	Green/Red ***	CONTRA	4
11	5.5	C2	0.17	MUA	324	LP/Inter	Inter	Red/Green ***	CONTRA	4

ID	Depth	Elec	RF	SUA/MUA	pk/trgh	SF	Speed	Color	Eye	Layer?
3	4	A2	~	MUA	381	~	~	Green/Red *	CONTRA	4/I
4	4	A2	~	MUA	468	~	~	Green/Red **	CONTRA	4/I
7	4.5	A2	0.91	SUA	320	LP	Fast	~	~	3
8	4.5	A2	0.56	SUA	282	Inter	Inter	~	~	3
9a	4.5	A2	~	MUA	305	~	~	Green On #	IPSI	3
9b	4.5	A2	0.17	MUA	418	Inter	Inter	~	~	3
12	5.5	A2	0.73	MUA	298	LP	Fast	No Tuning	IPSI	2
13	5.5	A2	0.62	SUA	245	LP	Fast	On/Off ****	IPSI	2

Table S1 | Properties of each unit from Patient #2. The three tables show all units (upper table) and the units obtained from the central electrode (middle table) and anterolateral electrode (lower table) separately, illustrate the progression through the LGN, from dorsal toward ventral layers. The RF column indicates the r-squared value from the forward model, red squares indicate good fits (r-squared>0.5). pk/trgh is the peak to trough time of the average waveform in µs. SF: spatial frequency tuning, LP: low-pass (responds to low spatial frequencies), Inter: Intermediate with high responses at low and intermediate spatial frequencies. For color-tuning the asterisks indicate the significance of the comparison between two phases of the checkerboard (independent samples t-test, #:

trend, *:$p<0.05$, **:$p<0.01$: ***:$p<0.001$, Bonferroni correction applied). The ‘Layer’ column shows our estimate of the most likely layer of the unit, taking the measurements of all units into consideration.

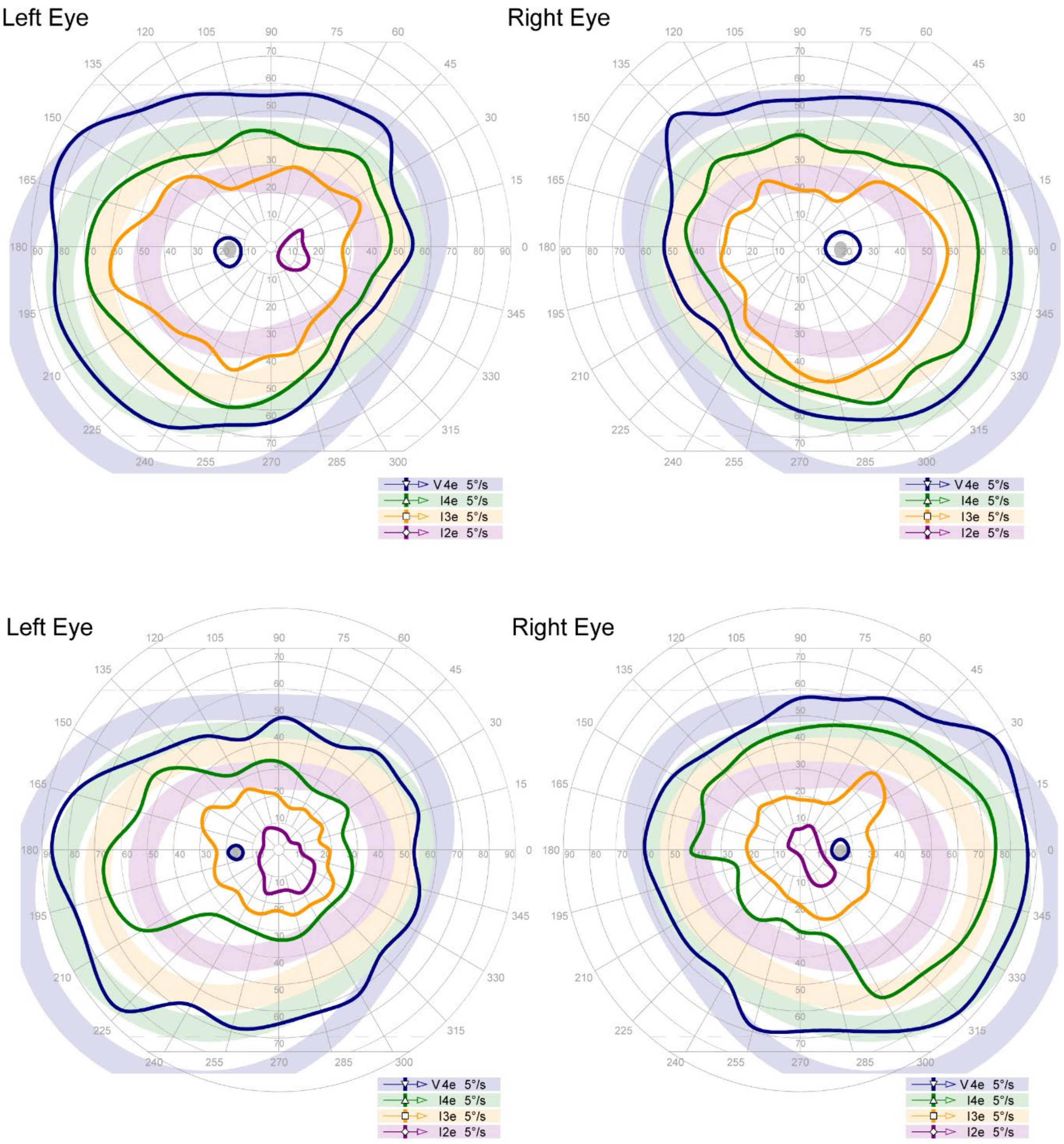

Figure S1 | Campimetry. Post-surgical kinetic perimetry results from Patient #1 (top row) and Patient #2 (bottom row). Perimetry was performed with an Octopus 900 system (Haag-Streit) with a stimulus moving at 5°/s. The different colors indicated the visual field sensitivity at different luminance and size values (Size V: $64mm^2$, Size I: $0.25mm^2$. Luminance 2-4: 100-1000 apostilibs (1 apostilib = $1/\pi$ $cd.m^{-2}$)). The blind-spot is indicated in blue. Normal ranges are indicated by the shaded regions.

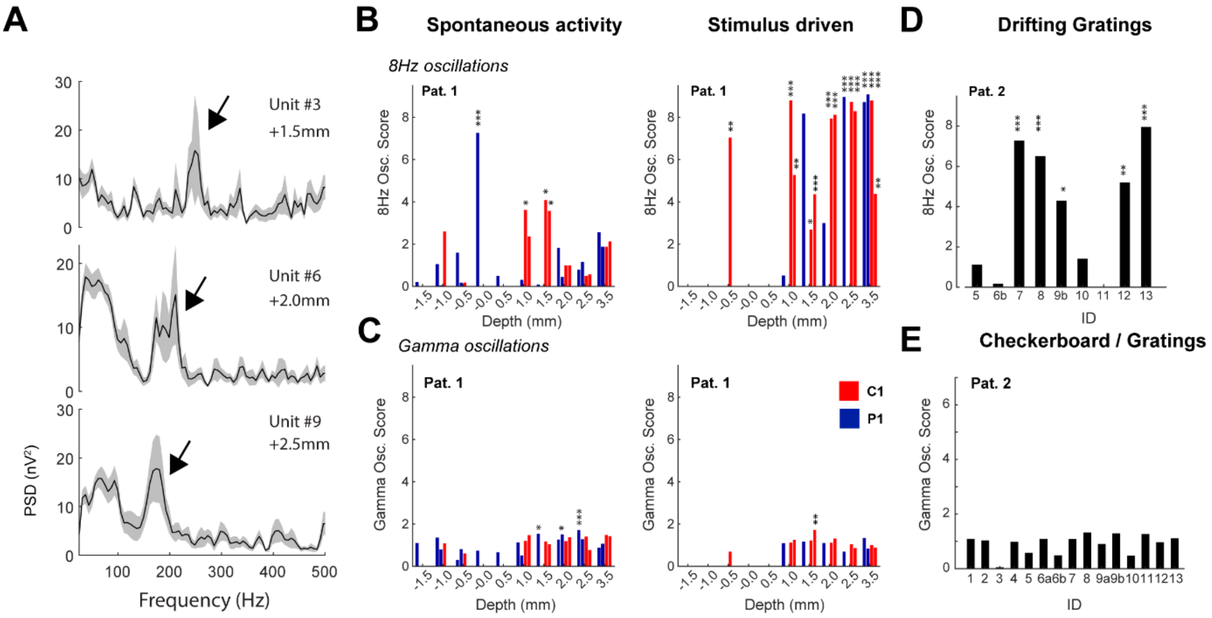

Figure S2 | Bursting responses in Patient 1 and gamma oscillations in Patients 1 and 2. A. Power spectral density function of spiking responses from three single units in Patient #1 in response to 8Hz flickering stimuli. High-frequency bursting responses are indicated by the arrows. We defined burst-spikes as those with inter-spike intervals less than 6.7ms (i.e. > 150Hz) and found that between 32% and 59% of spikes were fired in bursts during 8Hz flicker stimulation. **B-E.** Measures of oscillatory spiking in both patients**.** We calculated two oscillation scores. **B**. 8Hz Oscillation score in Patient 1. We calculated the ratio of the maximum power spectral density (derived from spiking auto-correlograms) between 7-9Hz and the power between 5Hz and 25Hz during spontaneous activity (left panel) and during an 8Hz flickering light (stimulus driven, right panel). Red bars come from the central electrode C1, blue bars from the posterior electrode P1. Different bars at the same depth come from different units clustered off the same electrode. **C.** The gamma-frequency oscillation score, which has been used previously[1], defined as the maximum power spectral density between 30-90Hz divided by the mean power between 10-120Hz. The values were close to 1, which implies the absence of peaks in the gamma band. The oscillation scores were considerably lower than the values of ~6 observed under halothane anesthesia in cats[1]. Asterisks indicate oscillatory scores significantly larger than 1 (***: p < 0.001, **: p < 0.01, *: p<0.05, not corrected for multiple comparisons). Significance was based on a comparison to a null distribution, which was derived by shuffling spike-times. Specifically, the same number of spikes were generated at random time-intervals (uniform distribution) between the timepoint of the 1st and last spike of the recording session. The p-value is the proportion of oscillation scores generated from 1,000 shuffled spike-trains that were greater than or equal to the calculated oscillation score. **D.** The 8Hz oscillation score measured during presentation of 8Hz drifting gratings in patient 2 (not tested for units 1-4, 6a and 9a). **E.** The gamma oscillation score measured during the presentation of high-contrast checkerboard presentation (units 1-4, 6a and 9a) or drifting gratings (other units) in patient 2.

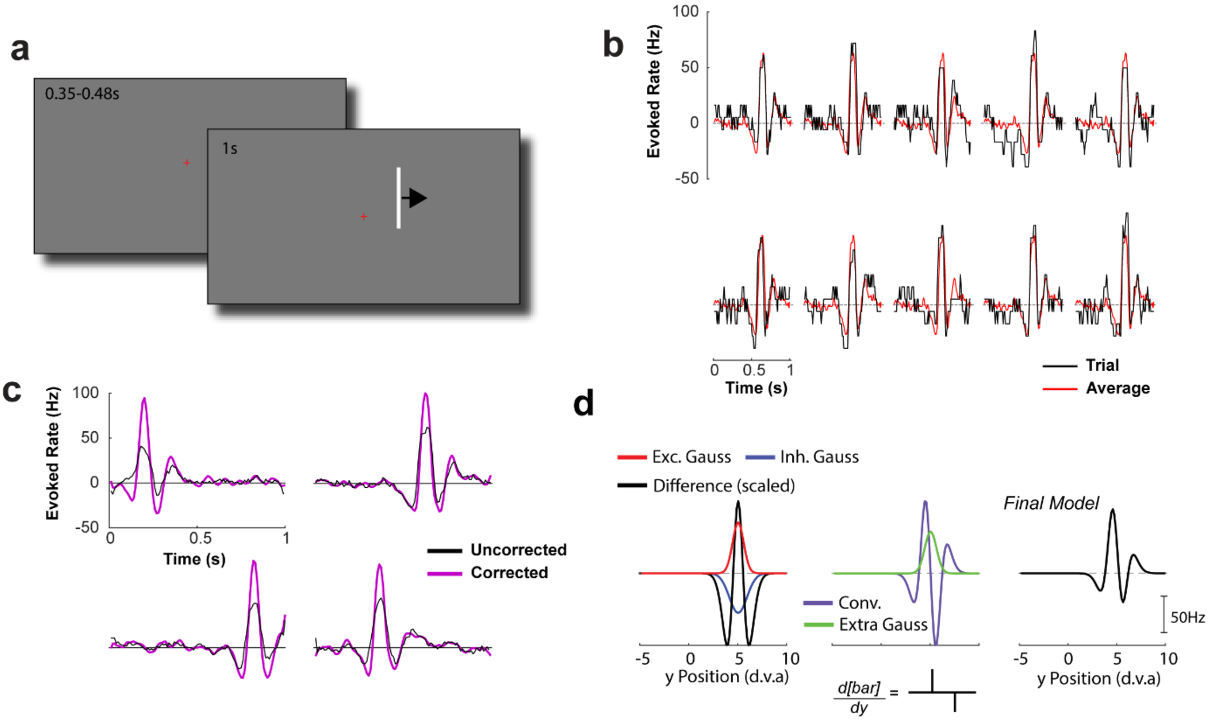

Figure S3 | Receptive field mapping Method. a, Receptive fields were mapped with a moving (15 deg./s) white bar on a grey background. The patient was trained on the day prior to the operation to maintain fixation on a central fixation dot using an eye-tracker (see Methods) but for technical reasons the patients' eyes could not be tracked during the surgery. **b,** The neuronal responses to moving bars were highly consistent across trials, which implies successful fixation. The graphs show the firing rate of Unit #7 in Hz on ten trials (black traces) elicited by rightwards bar sweep, after subtraction of the baseline firing rate. The red trace shows the average response across ten trials. **c,** To correct for small shifts of the eye across trials, we shifted time-axis of the responses of single trials based on the cross-correlation between the single trial response and the average response. The four graphs show the mean PSTHs to four directions of the bar before (black trace) and after correction (purple trace). **d,** The difference-of-Gaussians model used to fit the responses to the drifting bar. The model of the dynamic response consists of an excitatory and inhibitory Gaussian (red and blue in the left panel), which were convolved with the first differential of the luminance profile of the bar stimulus (purple line). We modeled the stationary component with an additional Gaussian (green).

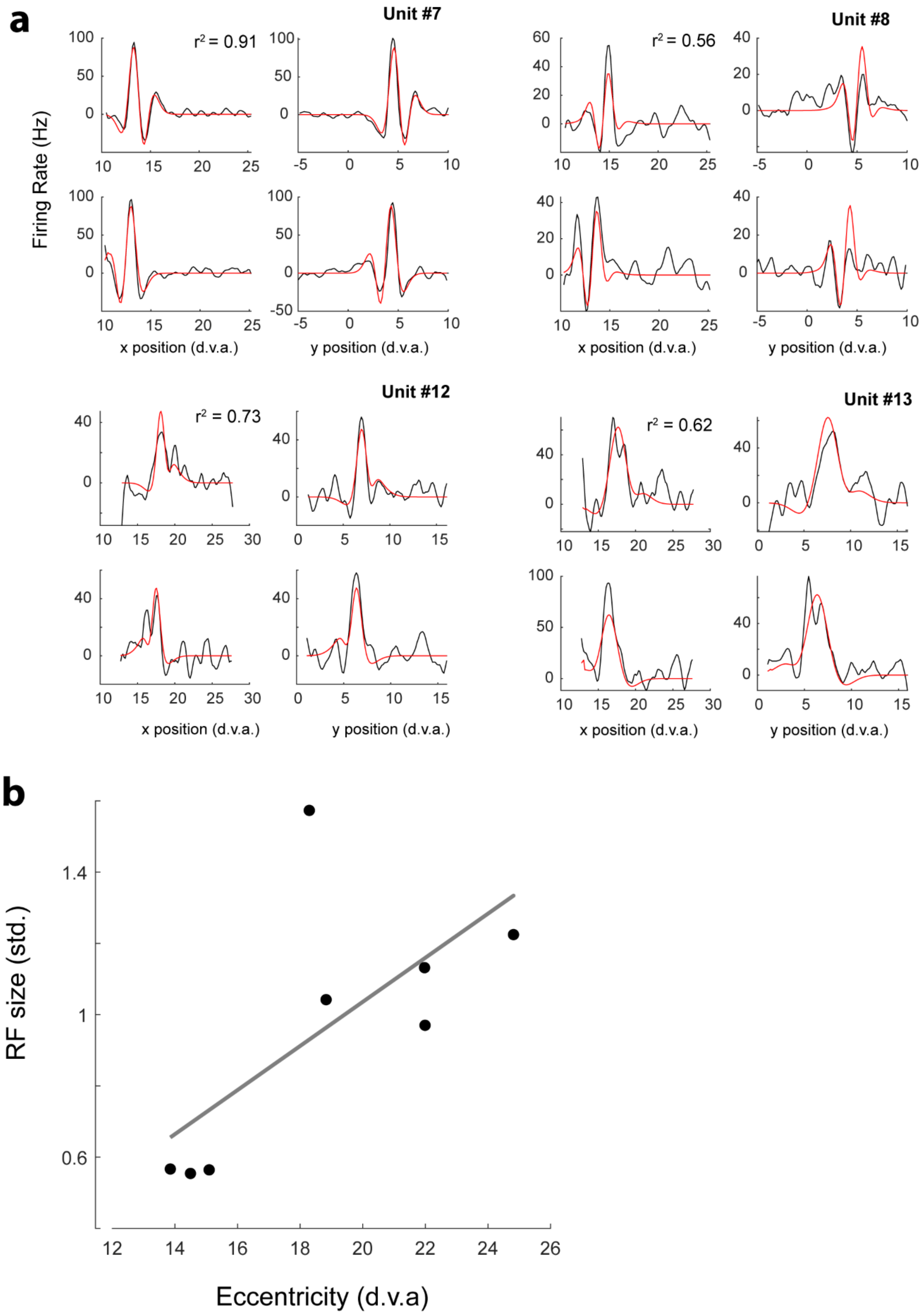

Figure S4 | Receptive field fits. a, Fits in the format of Figure 2c for four units with an r^2 value of greater than 0.5.**b,** Relationship between the eccentricity of the RF and the size of the excitatory sub-unit (standard deviation), all units with positive r^2 values are shown.

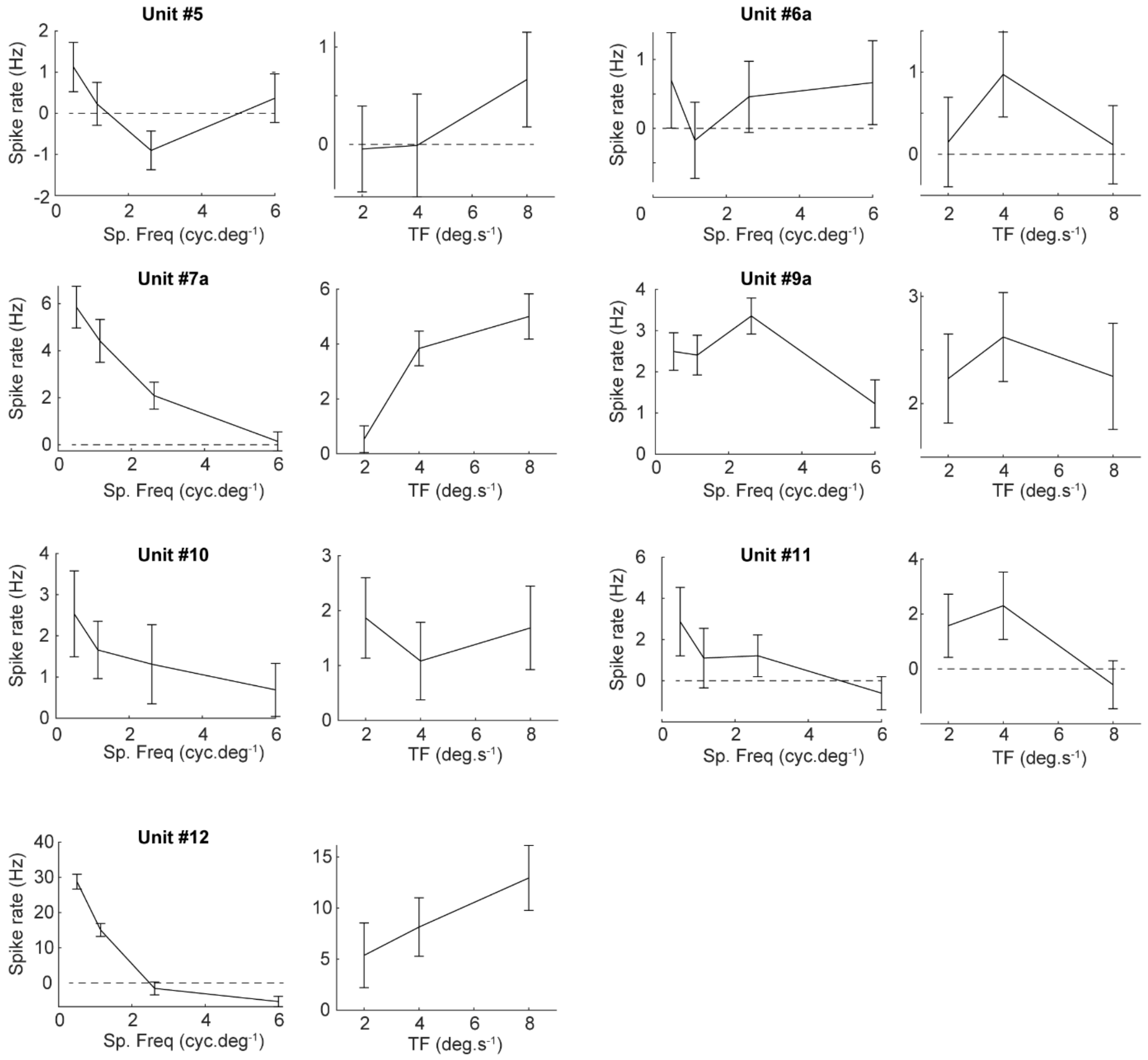

Figure S5 | Tuning to spatial frequency and speed. Spatial and temporal frequency tuning curves of all units not reported in Figure 2. Error bars are s.e.m. across trials.

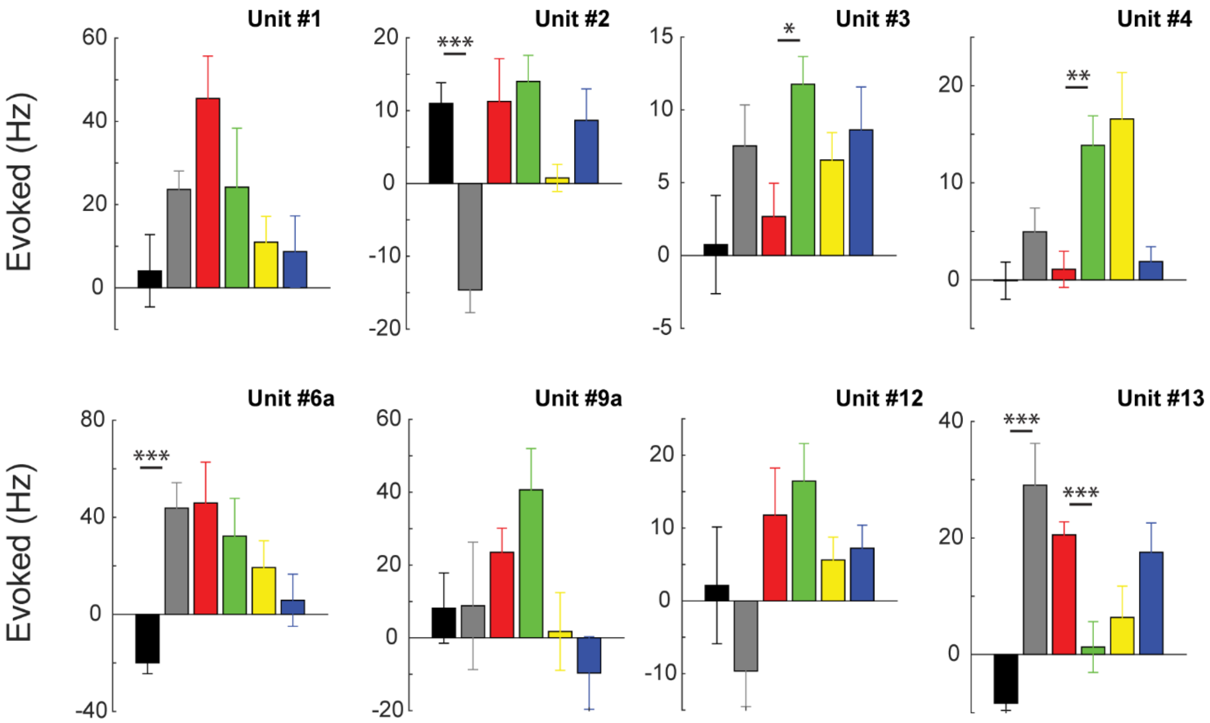

Figure S6 | Color tuning. (a-c), Evoked (baseline corrected) responses of all units to colored checkerboards. Format as in Figure 2h. * = $p<0.05$, ** =$p<0.01$, ***=$p<0.001$, all independent samples t-tests with Bonferroni multiple comparison correction applied.

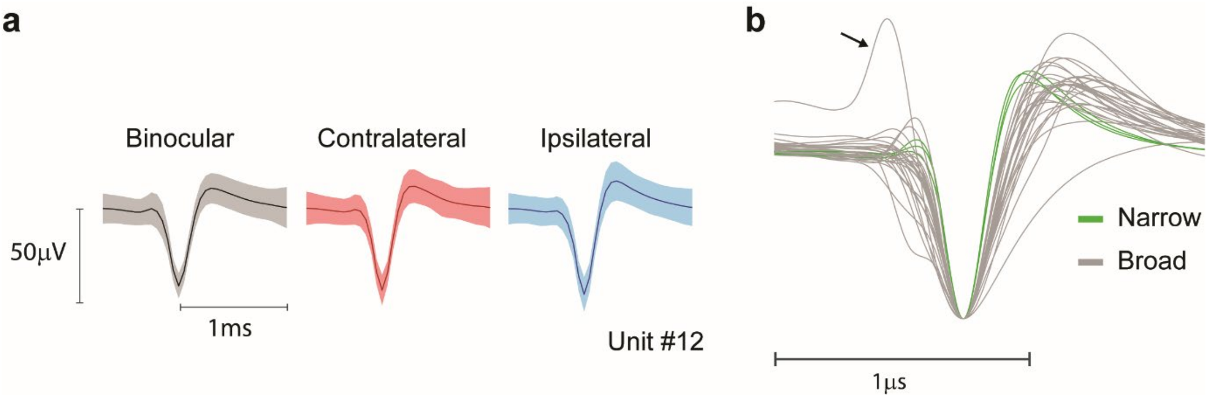

Figure S7 | Waveforms. a, Average spike waveforms from Unit #12 during binocular and monocular viewing. The spike waveform remained stable during these viewing conditions indicating that changes in spontaneous firing rates are unlikely to be due to changes in spike-isolation. **b,** Average waveforms from all 30 units from both patients. The green colors mark narrowest waveforms (Units #5, #10, #13) with a full-width half-maximum of the initial peak of less than 150μs and a peak-trough distance of less than 250μs. Spikes are shown normalized to the maximum amplitude. One unit (#8) had a biphasic positive first response (arrow).
