# Supplementary material for: Effects of eye closure on the spiking activity of human lateral geniculate neurons
Source: Nat Commun. 2025 Nov 24;16:10402. doi: 10.1038/s41467-025-65383-x (PMC12645034; doi:10.1038/s41467-025-65383-x)
Supplement: Supplementary file 3 — Description of supplementary [file 41467_2025_65383_MOESM3_ESM.pdf]

nature portfolio

Peer Review File

Effects of eye closure on the spiking activity of human lateral geniculate neurons

Corresponding Author: Professor Pieter Roelfsema

This file contains all reviewer reports in order by version, followed by all author rebuttals in order by version.

Version 0:

Reviewer comments:

Reviewer #1

(Remarks to the Author)

Authors present a rare dataset of recordings from the human LGN as part of an experimental epilepsy treatment from individuals with photo sensitive epilepsy, one of which was related to an intrinsic glioma that was resected.

The recordings were made using DBS-type microelectrodes with a trajectory that captured some layer specific data potentially. There are two subjects. From what I can ascertain, maybe 6 units were isolated as true single unit activity and the remainder was multi unit activity. These are rare data and include some fascinating empirically information such as the estimated response latency that is highly robust even with limited numbers of neurons. The authors should be commended on taking advantage of this opportunity with a fairly sophisticated set of analyses that make the most of limited underlying data. I think this represents a unique contribution to the literature for the human visual system.

The eye closure data are interesting and assessed using robust methods in my opinion. The two units shown in Figure 3 are presented fairly convincingly. I thought the controls for the factor of time were well constructed.

The layer assignments are performed based on behavioral characteristics principally. I understand histology is impossible. Can the authors perform these layer assignments using a model fit that takes into account the OCIX, color properties, and spatial tuning? That might make these data more convincing given the limitations. Readers could then understand how each neuron's assignment makes sense relative to alternative assignments of layer.

The hypotheses being tested here are slightly ambiguous, but perhaps one of the main objectives was to test for the presence of direction sensitive neurons which is cited as a main controversy in primate visual systems. Given this as a principal focus, the authors can perhaps expand upon these results.

In Figure S6, why is it that the significant units do not appear to have different values than the non-significant units? Also, for these critical neurons which are testing a specific, controversial prediction of the existing human/monkey literature, is it possible to show that the directional tuning is significantly greater than spatial tuning? I think unit 13 is included as both spatial location an directional tuning, which seems like a potential confound for directional tuning. Perhaps for this key analysis the authors should show that removing variance explained by location does not eliminate significance for directional tuning.

The significance for spatial tuning excludes the 6Hz data, but I am not sure the curves suggest the responses are obviously weaker at this frequency. If this frequency was too fast, then why was it included? Also, the significance for directional tuning uses a null of 10k shuffles, which seems like a large number given the small number of trials, which would then inflate the p value. Are the results consistent with a more reasonable number of shuffles, like 1000?

In the table describing waveforms, can the authors include the statistical results of the tests for specific tuning properties for each neuron? It probably wouldn't make a difference given the magnitude of p values, but do the authors need to correct for the different tuning tests applied to each neuron?

The analysis of spike width for neuron subtype is reasonable, though limited by the number of neurons to generate a

distribution of widths etc. Given that conclusions about inter neuron identity are predicated on analogy to cortical neurons, the authors should use datasets of cortical neurons in the spike width distributions to understand how the recorded thalamic neurons fit with characteristics of interneurons/pyramidal neurons observed in larger datasets. This will allow them to statistically test for the width values relative to distributions of more definitively categorized neuron types.

How much did the fixation shift for each trial? Can this be estimated from the differences in the preferred spike location?

Reviewer #2

(Remarks to the Author)
GENERAL COMMENTS
Self et al recorded visual responses in the lateral geniculate nucleus (LGN) of two human patients who were undergoing surgery for epilepsy in the occipital lobe. The strength and novelty of the study is that the recordings are in human LGN, for which we have no data. Unfortunately, however, and understandably (given the time constraint of the surgery) the data is essentially anecdotal, as it is based on a handful of cells. As such it is difficult to make any meaningful conclusion from the study. All that one can conclude is that there are at least some cells (4-5) in the human LGN that look somewhat like monkey LGN cells. I am not sure how this weakness can be improved given the inability to go back and collect more data or, I am guessing, the difficulty of recording from additional patients. I am providing a few suggestions below.
The presentation could also be improved by providing more context for this study and a better comparison with existing literature in the non-human primate. The manuscript lacks a discussion section that could help the reader.

MAJOR SPECIFIC COMMENTS
1. Essentially all the data on receptive fields (RFs) comes from a single patient (#2). The authors recorded 13 cells from this patient, but the data in the figures are presented as individual example neurons. The entire manuscript is based on data from 4-5 cells. It would improve the manuscript if some population analysis could be done across all 13 cells. For, example distribution of SF and TF preferences and tuning, etc.
2. Given the low number of recorded cells, data for each individual neuron should be shown.
3. There is no data on RF size, the authors mapped response fields with bars, what is the average RF size of neurons at the recorded eccentricities and how does this compare to macaque RF sizes? The one example neuron shown in Fig. 2d seems to have a much larger RF size than in macaque LGN for example.
4. The results in Fig. 3 are potentially interesting, but are based on 2 putative inhibitory cells. We have no idea whether these are truly inhibitory cells as, as also acknowledged by the authors, there is no data relating spike waveform to cell types in LGN. Moreover, the neurons classified as inhibitory in this study had pk/trgh values ranging from 223-245µs, which, in Mitchell et al 2007 were classified as excitatory cells (their criterion for inhibitory neurons was <200µs).
5. The paper is lacking a necessary Discussion section relating the results of the study with previous literature in monkeys. How does RF size, SF, TF, color etc tuning compares with that of previous macaque studies?
6. Results Lines 58-59. "…spiking was apparent from +2mm to +6mm." It really seems from +0.5 mm to +6.6 mm.
7. The data on orientation tuned cells is weak, as also acknowledged by the authors. It is based on 3 cells of which one is a multi-unit, with very low firing rates and small differences in firing rates between preferred and non-preferred stimuli, as well as coarse sampling of orientations.
8. Many figure panels report "evoked activity" instead of firing rates. Define evoked activity.
9. Lines 124-125. In FigS8 please show the waveform of the two units deemed to be inhibitory (#10-13).
10. Methods line 361. Specify what anesthetic was used for general anesthesia and at what dose (we know the type and depth of anesthesia affects electrophysiological recordings and neuronal responses).

MINOR COMMENTS

1) Line 65. Spell d.v.a. out the first time it is used.
2) Line 101. Reference 19 is from mouse so inappropri

Reviewer #3

(Remarks to the Author)
In this study, Self and colleagues report the responses of single unit neurons in the human lateral geniculate nucleus to visual inputs. This is an extremely rare and valuable dataset, as these recordings are not normally captured through the course of intracranial surgery for epilepsy. In these cases, the clinical team recommended placement of DBS electrodes in the LGN for neuromodulation due to the visual nature of their seizures. During the DBS procedure, the authors also collected single unit spiking activity through the use of microelectrodes used to map the borders of the LGN. During the awake operative procedure, the study team presented visual stimuli (gratings, checkerboards, etc) while recording spiking activity. This is an interesting and potentially highly valuable study, as this would represent the first report of such activity in the human brain to my knowledge. The manuscript is largely descriptive in nature, mostly characterizing the responses of these neurons and demonstrating, as expected, some of the response heterogeneity. As such, there are not many major criticisms or suggested revisions. However, there are some slightly less critical points that could help with the overall conclusions of the manuscript.

The major claim of the study is that the responses to single eye closing are different among different neurons. This motivates the interpretation that some neurons in the ipsilateral LGN because they may be inter-neurons, which is based on their more relatively narrow waveform. It is hard to be sure of this conclusion, given the relatively small sample size of neurons exhibiting this behavior. The authors have tempered this claim somewhat, but perhaps even more caution should be exercised here. Nonetheless, it is an interesting result. One thing that would certainly make this more accessible to the general readership is that, when looking at the figures, it is often hard to keep track of what response is ipsilateral versus contralateral to the closed eye. The authors show an image of two eyes, one closed one open (left and right), but perhaps they could also indicate somehow there the ipsilateral or contralateral eye to the neuron being shown is the one being closed.

It also appears that when a neuron is changing its activity during eye closure, whether the apparent decreases in activity are significantly lower than the eyes open condition. This is certainly true when the subject is viewing a black screen, and so the spontaneous activity is lower. But it appears that the response to the white screen is almost as strong as the eyes open condition? In this case, is there a meaningful difference between the two sides.

Finally, the authors also provide a model in Figure 3 summarizing their findings, which is a nice illustration regarding the different types of responses they observe. The only concern with this model is the layers of LGN that they estimate. It is not clear how these estimates are generated, aside from using the depth of the microelectrode and comparing the relative depths. Even so, however, there could be several factors that would make the direct translation from depth to LGN layers challenging.

Reviewer #4

(Remarks to the Author)
Using a unique patient opportunity, the investigators recorded SUA from the LGN. Compared to the existing neurophysiology work in monkey LGN, the human LGN cells show remarkably similar receptive fields, sensitivity to color, and orientation tuning. Volitional eye closures modulated evoke unit activities, with enhancement and inhibition of firing rate, providing additional functional granularity in humans. While these findings offer powerful cross-species alignment of the LGN neurophysiology, they don’t provide significant conceptual insights into the LGN function. A major limitation is that a single subject provided the receptive field and tunning information, as the first patient provided coarse functional assessments with a flashing light. There were also a limited number of well-isolated neurons and many multi-unit responses showing intermediate effects. There are also no trial-by-trial roster plots showing within-subject response consistency. Finally, given the sparse sampling and contentious role of the spike-wave shapes, assigning cell layers to unit responses might be premature.

Version 1:

Reviewer comments:

Reviewer #1

(Remarks to the Author)
The authors have done a reasonable job responding to the critiques and acknowledging the limitations of the study. They also removed what I thought was the most controversial analysis.

The only suggestion I have is to clarify in the Discussion how their findings bear upon stated controversy in conceptions of perception, as they introduce this concept in the introduction to motivate the study.

Reviewer #2

(Remarks to the Author)
The authors have revised the manuscript as much as it was possible with the limited dataset.
I still feel there is very little new information we have gained from this study, but it is more than zero.

Reviewer #3

(Remarks to the Author)

The authors have addressed the concerns raised by the first round of review to the extent possible given the limited dataset presented. As noted in the reviews, this is largely a descriptive study reporting the results of LGN recordings in two patients. This would still be a valuable addition to the literature, as these data do not exist. The authors have sufficiently revised the manuscript to improve the overall presentation of the results.

Reviewer #4

(Remarks to the Author)
In their original submission, the authors demonstrated that LGN cells respond to voluntary eye closure. In this revision, they

have clarified the theoretical importance of this finding, highlighting its implications for ocular competition, binocular rivalry, and ocular gain control—questions that are difficult to address in non-human primates. The inclusion of raster plots and single-trial analyses enhances confidence in the reliability of the responses, and the layer assignment is now further clarified, with expanded discussion providing greater transparency.
Although the sample size remains limited due to the rarity of such recordings, the authors make a strong case that their observations provide unique insights into human LGN physiology. The identification of previously undescribed changes in spontaneous activity and unit-specific increases during eye closure extends the conceptual contribution of the study. Overall, the revisions strengthen the manuscript, and I am satisfied that my concerns have been fully addressed.

Open Access This Peer Review File is licensed under a Creative Commons Attribution 4.0 International License, which permits use, sharing, adaptation, distribution and reproduction in any medium or format, as long as you give appropriate credit to the original author(s) and the source, provide a link to the Creative Commons license, and indicate if changes were made.
In cases where reviewers are anonymous, credit should be given to 'Anonymous Referee' and the source.
The images or other third party material in this Peer Review File are included in the article's Creative Commons license, unless indicated otherwise in a credit line to the material. If material is not included in the article's Creative Commons license and your intended use is not permitted by statutory regulation or exceeds the permitted use, you will need to obtain permission directly from the copyright holder.
To view a copy of this license, visit https://creativecommons.org/licenses/by/4.0/

We would like to thank all the reviewers for their careful reading of the manuscript and their constructive critique. We have extensively revised the manuscript to address all concerns and the changes to the manuscript are shown in red text in the 'tracked changes' version. Based on the feedback, we have improved and expanded the introduction and discussion sections. Furthermore, we now included data from all units in the supplementary information. We believe the revised manuscript addresses all of the reviewers' concerns.

Reviewers' comments:

Reviewer #1 (Remarks to the Author):

Authors present a rare dataset of recordings from the human LGN as part of an experimental epilepsy treatment from individuals with photo sensitive epilepsy, one of which was related to an intrinsic glioma that was resected.

The recordings were made using DBS-type microelectrodes with a trajectory that captured some layer specific data potentially. There are two subjects. From what I can ascertain, maybe 6 units were isolated as true single unit activity and the remainder was multi unit activity. These are rare data and include some fascinating empirically information such as the estimated response latency that is highly robust even with limited numbers of neurons. The authors should be commended on taking advantage of this opportunity with a fairly sophisticated set of analyses that make the most of limited underlying data. I think this represents a unique contribution to the literature for the human visual system.

We thank the reviewer for their careful reading of the paper and their positive comments.

The eye closure data are interesting and assessed using robust methods in my opinion. The two units shown in Figure 3 are presented fairly convincingly. I thought the controls for the factor of time were well constructed.

The layer assignments are performed based on behavioral characteristics principally. I understand histology is impossible. Can the authors perform these layer assignments using a model fit that takes into account the OCIX, color properties, and spatial tuning? That might make these data more convincing given the limitations. Readers could then understand how each neuron's assignment makes sense relative to alternative assignments of layer.

We have now provided an extended description of the layer assignments in the revised Discussion section. Layer assignments were made by considering all measured tuning properties of the unit: ocularity, preferred spatial and temporal

frequency, and color-tuning. In addition, we considered the depth of the unit along the electrode trajectory and, in cases where the assignment was still uncertain, we used interpolation. For example, Unit #5 could not be unambiguously assigned to a layer based on its RF characteristics. However, units located more dorsal and ventral relative to this unit were estimated to be located in layers 6 and 4, respectively, and multi-unit activity at the same depth mainly responded to the ipsilateral eye, making it very likely that unit #5 was in layer 5.

The hypotheses being tested here are slightly ambiguous, but perhaps one of the main objectives was to test for the presence of direction sensitive neurons which is cited as a main controversy in primate visual systems. Given this as a principal focus, the authors can perhaps expand upon these results.

The main hypothesis we set out to test was whether human LGN neurons have properties that resemble those in monkeys or whether there are key differences. Our observations largely support the idea that human LGN cells resemble those in monkeys. We have expanded the introduction and discussion to clarify how our data contribute to the translation of knowledge from animal work to humans. We reanalysed all the data including the direction-tuning data. Because the number of trials per direction was small, the data were around the threshold of significance. We have removed these data to prevent overinterpretation.

In Figure S6, why is it that the significant units do not appear to have different values than the non-significant units? Also, for these critical neurons which are testing a specific, controversial prediction of the existing human/monkey literature, is it possible to show that the directional tuning is significantly greater than spatial tuning? I think unit 13 is included as both spatial location an directional tuning, which seems like a potential confound for directional tuning. Perhaps for this key analysis the authors should show that removing variance explained by location does not eliminate significance for directional tuning.

As was mentioned in response to the previous point, the low number of trials per direction that were tested prompted us to remove this analysis.

The significance for spatial tuning excludes the 6Hz data, but I am not sure the curves suggest the responses are obviously weaker at this frequency. If this frequency was too fast, then why was it included?

We assume here that the reviewer is referring to the condition with gratings with a spatial frequency of 6 cyc/deg, which we excluded from our analysis for two reasons. Firstly, this high spatial frequency produced very weak responses across all units (see Figure S5a,d,g). During our preparations, we decided to include this condition because some LGN cells in monkeys respond to such high spatial frequencies, but only if they have RFs close to the fovea. The cells recorded by us had more eccentric RFs and they preferred lower spatial frequencies. Secondly, the LCD

screen’s pixel resolution in the operating theatre did not optimally display the 6 cyc/deg stimulus and the effective contrast of the stimulus was lower than for the other gratings. In the revised manuscript we explain why we excluded of the 6 cyc/deg condition.

Also, the significance for directional tuning uses a null of 10k shuffles, which seems like a large number given the small number of trials, which would then inflate the p value. Are the results consistent with a more reasonable number of shuffles, like 1000?

This comparison has been removed from the paper, as has been explained in our responses to the previous points.

In the table describing waveforms, can the authors include the statistical results of the tests for specific tuning properties for each neuron? It probably wouldn’t make a difference given the magnitude of p values, but do the authors need to correct for the different tuning tests applied to each neuron?
We have applied a Bonferroni correction for multiple comparisons for all tests reported in the manuscript. We have added p-values to the various tests of tuning properties in Table S1.

The analysis of spike width for neuron subtype is reasonable, though limited by the number of neurons to generate a distribution of widths etc. Given that conclusions about inter neuron identity are predicated on analogy to cortical neurons, the authors should use datasets of cortical neurons in the spike width distributions to understand how the recorded thalamic neurons fit with characteristics of interneurons/pyramidal neurons observed in larger datasets. This will allow them to statistically test for the width values relative to distributions of more definitively categorized neuron types.

We have now expanded the number of neurons included in the spike-width analysis by including all units from Patient #1 in the distribution as well. These data are now shown in Figure 4i and Figure S7. It is clearer that the narrow-wave shape cells have exceptionally narrow spike widths in this expanded data set. The sample is still too small to test for bimodality of the distribution, which we now acknowledge in the legends of Fig. 4i and in the discussion (section on spontaneous activity changes during voluntary eye closure). We also included a comparison to the spike-widths observed in cortical/hippocampal studies as suggested by the reviewer (see the section on spike-sorting in Methods).

How much did the fixation shift for each trial? Can this be estimated from the differences in the preferred spike location?

This is a great suggestion. We were able to estimate the amplitude of fixation shifts for cells with clearly mapped RFs. Our estimates suggest that the variations in gaze position have a standard deviation of 0.44 d.v.a. in the x direction and 0.21 d.v.a. in the y-direction. These values are now reported on Lines 576-578.

Reviewer #2 (Remarks to the Author):

GENERAL COMMENTS
Self et al recorded visual responses in the lateral geniculate nucleus (LGN) of two human patients who were undergoing surgery for epilepsy in the occipital lobe. The strength and novelty of the study is that the recordings are in human LGN, for which we have no data. Unfortunately, however, and understandably (given the time constraint of the surgery) the data is essentially anecdotal, as it is based on a handful of cells. As such it is difficult to make any meaningful conclusion from the study. All that one can conclude is that there are at least some cells (4-5) in the human LGN that look somewhat like monkey LGN cells. I am not sure how this weakness can be improved given the inability to go back and collect more data or, I am guessing, the difficulty of recording from additional patients. I am providing a few suggestions below.
The presentation could also be improved by providing more context for this study and a better comparison with existing literature in the non-human primate. The manuscript lacks a discussion section that could help the reader.

We thank the reviewer for their useful comments. We agree with the reviewer that the sample size is small and cannot be increased. However, we would like to note that several of our findings do not require large sample sizes, for example we demonstrate clear red-green opponent responses in single-neurons in human LGN. This is an important confirmation of findings in the primate literature going beyond anecdotal. Furthermore, our findings on the effects of eye closure on spontaneous activity are novel and the effects are large and remarkable. Hence, these findings increase our knowledge of LGN activity and will stimulate further experiments. We agree with the reviewer that our discussion was too short and we have now expanded it.

MAJOR SPECIFIC COMMENTS
1. Essentially all the data on receptive fields (RFs) comes from a single patient (#2). The authors recorded 13 cells from this patient, but the data in the figures are presented as individual example neurons. The entire manuscript is based on data from 4-5 cells. It would improve the manuscript if some population analysis could be done across all 13 cells. For, example distribution of SF and TF preferences and tuning, etc.

We agree that a more systematic evaluation across the population would improve the paper. Prompted by the reviewer, we now added the spatial and temporal frequency tunings from all cells to Figure S5 and color tunings from all cells to Figure S6. The revision also provides the new Supplementary Figure S4 with RF sizes and their relationship with eccentricity.

2. Given the low number of recorded cells, data for each individual neuron should be shown.

We have now included data from all cells in the paper in the supplementary figures.

3. There is no data on RF size, the authors mapped response fields with bars, what is the average RF size of neurons at the recorded eccentricities and how does this compare to macaque RF sizes? The one example neuron shown in Fig. 2d seems to have a much larger RF size than in macaque LGN for example.

We have now extensively revised our reporting and discussion of RF sizes. The reviewer is correct that the sizes reported by us are larger than those reported in the macaque. This larger size is likely the result of both the higher eccentricity of the RFs compared to those typically reported in the monkey literature, and the fact that the large majority of LGN RF sizes in macaques were recorded in anesthetized, paralysed animals. We have added the following text to the discussion section:

"The RFs studied by us were larger than those recorded in non-human primates. These earlier studies mapped RF sizes by hand, or reported the radius of the central sub-unit from a difference-of-Gaussians (DoG) fit to the responses to drifting gratings of different spatial frequencies[6]. We here estimated RF size using drifting bars, which is more time-efficient and also allows the fitting of a DoG model. The best-fitted RF of the present study had an eccentricity of 13.8 degrees and was likely parvocellular. The central sub-unit was 0.57 deg, which is larger than the values of 0.06-0.15 degrees that were measured at this eccentricity in anesthetized, paralyzed monkeys[6,27]. It is conceivable that we overestimated sizes of the RFs because the subject made small eye-movements.".

We have also included population data showing the relationship between RF size and eccentricity as Figure S4b.

4. The results in Fig. 3 are potentially interesting, but are based on 2 putative inhibitory cells. We have no idea whether these are truly inhibitory cells as, as also acknowledged by the authors, there is no data relating spike waveform to cell types in LGN. Moreover, the neurons classified as inhibitory in this study had pk/trgh values ranging from 223-245μs, which, in Mitchell et al 2007 were classified as excitatory cells (their criterion for inhibitory neurons was <200μs).

We have expanded the number of spike-widths measured by including all units from Patient #1 in our sample, as was mentioned in our reply to one of the previous points. Of the 30 units recorded by us, none had spike-widths less than 200μs and we classified the narrowest spikes in our population of LGN units as putative inhibitory neurons. It is conceivable that human LGN neurons have broader spike-widths than cortical neurons in monkeys. However, we agree with the reviewer that it

is impossible to determine whether these cells were truly interneurons, and we are careful not to overstate that claim in the manuscript. We now state: “In the cortex interneurons have the narrowest wave-shapes, raising the intriguing possibility that these cells were interneurons. The shortest peak-to-trough times in our sample (223 and 245μs) were somewhat longer than the values of cortical interneurons[26] (150-200μs), but little is known about action potential waveshapes in the human thalamus.”

5. The paper is lacking a necessary Discussion section relating the results of the study with previous literature in monkeys. How does RF size, SF, TF, color etc tuning compares with that of previous macaque studies?

Thanks for your advice. We have now added an improved discussion section to the manuscript with comparisons to the monkey literature.

6. Results Lines 58-59. “…spiking was apparent from +2mm to +6mm.” It really seems from +0.5 mm to +6.6 mm.

Thanks for your observation. We have changed the text accordingly.

7. The data on orientation tuned cells is weak, as also acknowledged by the authors. It is based on 3 cells of which one is a multi-unit, with very low firing rates and small differences in firing rates between preferred and non-preferred stimuli, as well as coarse sampling of orientations.

We agree that this data is weak because orientations were sampled too coarsely to adequately assess tuning. We have removed the orientation/direction tuning from the paper.

8. Many figure panels report “evoked activity” instead of firing rates. Define evoked activity.

Evoked activity (this is simply baseline corrected activity) is now defined on line 598.

9. Lines 124-125. In FigS8 please show the waveform of the two units deemed to be inhibitory (#10-13).

They are now highlighted in green in the figure (Fig. S7 of the revision).

10. Methods line 361. Specify what anesthetic was used for general anesthesia and at what dose (we know the type and depth of anesthesia affects electrophysiological recordings and neuronal responses).

We think there is a misunderstanding here. The recordings were made in the awake state, only a local anesthetic was used on the scalp. General anesthesia was only induced after conclusion of the recordings and placement of the DBS probe, when the DBS system and battery was placed in the chest and connected to the probe. The anesthetic used for general anesthesia is therefore not relevant to the recordings.

MINOR COMMENTS

1) Line 65. Spell d.v.a. out the first time it is used.

Corrected.

2) Line 101. Reference 19 is from mouse so inappropriate.

Removed

Reviewer #3 (Remarks to the Author):

In this study, Self and colleagues report the responses of single unit neurons in the human lateral geniculate nucleus to visual inputs. This is an extremely rare and valuable dataset, as these recordings are not normally captured through the course of intracranial surgery for epilepsy. In these cases, the clinical team recommended placement of DBS electrodes in the LGN for neuromodulation due to the visual nature of their seizures. During the DBS procedure, the authors also collected single unit spiking activity through the use of microelectrodes used to map the borders of the LGN. During the awake operative procedure, the study team presented visual stimuli (gratings, checkerboards, etc) while recording spiking activity. This is an interesting and potentially highly valuable study, as this would represent the first report of such activity in the human brain to my knowledge. The manuscript is largely descriptive in nature, mostly characterizing the responses of these neurons and demonstrating, as expected, some of the response heterogeneity. As such, there are not many major criticisms or suggested revisions. However, there are some slightly less critical points that could help with the overall conclusions of the manuscript.

We would like to thank the reviewer for their careful consideration of the manuscript sand their positive comments.

The major claim of the study is that the responses to single eye closing are different among different neurons. This motivates the interpretation that some neurons in the ipsilateral LGN because they may be inter-neurons, which is based on their more relatively narrow waveform. It is hard to be sure of this conclusion, given the relatively small sample size of neurons exhibiting this behavior. The authors have tempered this claim somewhat, but perhaps even more caution should be exercised here.

We agree with the reviewer and remain cautious in interpreting the data. We increased the sample of wave-shapes by including all units from patient #1 (Fig. S7b). The putative inhibitory cells had the narrowest wave-shapes out of 30 units, and only 3 of them had wave-shapes narrower than 250 microseconds. This increases the confidence that these cells were special.

Nonetheless, it is an interesting result. One thing that would certainly make this more accessible to the general readership is that, when looking at the figures, it is often hard to keep track of what response is ipsilateral versus contralateral to the closed eye. The authors show an image of two eyes, one closed one open (left and right), but perhaps they could also indicate somehow there the ipsilateral or contralateral eye to the neuron being shown is the one being closed.

We agree with the reviewer that it can be confusing to keep track of eye preference in the figures. All cells were recorded in the left LGN. Right eye stimuli were therefore contralateral (colour-coded red in the manuscript) and left eye stimuli ipsilateral (blue in the manuscript). We have extended this color-coding throughout Figure 4 to increase clarity. We have also now indicated which eye was preferred for each cell in the panels of Figure 4.

It also appears that when a neuron is changing its activity during eye closure, whether the apparent decreases in activity are significantly lower than the eyes open condition. This is certainly true when the subject is viewing a black screen, and so the spontaneous activity is lower. But it appears that the response to the white screen is almost as strong as the eyes open condition? In this case, is there a meaningful difference between the two sides.

We are not sure we fully understand this question. We estimated the spontaneous activity during the grey screen period prior to the appearance of a black/white checkerboard. The black and white dots in Figure 4e-g represent responses to different phases of the black-white checkerboard, but we did not present black or white screens. The responses to the different monocular stimulation conditions (left vs right eye) are shown in the improved Figure 4c and we also estimated the Ocularity Index. We have now added a Binocularity Index, which compares the activity elicited by monocular stimulation to that when both eyes were open (y-axis in Fig. 4c). Our analysis shows that the LGN responses to binocular stimulation were close to the monocular response elicited by the preferred eye.

Finally, the authors also provide a model in Figure 3 summarizing their findings, which is a nice illustration regarding the different types of responses they observe. The only concern with this model is the layers of LGN that they estimate. It is not clear how these estimates are generated, aside from using the depth of the microelectrode and comparing the relative depths. Even so, however, there could be several factors that would make the direct translation from depth to LGN layers challenging.

We have now added an extensive section to the discussion concerning the estimation of the layers. Layer assignments were made by considering all measured tuning properties of the unit, i.e. the ocularity, preferred spatial and temporal frequency, and color-tuning. In addition, we considered the depth of the unit along the electrode trajectory and, in cases where the layer was still uncertain, we used information from nearby units on the same electrode track. For example, Unit #5 could not be unambiguously assigned to a layer based on its RF characteristics. However, units located more dorsal and ventral to this unit were estimated to be

located in layers 6 and 4, respectively, and a multi-unit clustered from the same electrode at the same depth was mainly driven by ipsilateral eye, making it very likely that unit #5 was in layer 5.

Reviewer #4 (Remarks to the Author):

Using a unique patient opportunity, the investigators recorded SUA from the LGN. Compared to the existing neurophysiology work in monkey LGN, the human LGN cells show remarkably similar receptive fields, sensitivity to color, and orientation tuning. Volitional eye closures modulated evoke unit activities, with enhancement and inhibition of firing rate, providing additional functional granularity in humans. While these findings offer powerful cross-species alignment of the LGN neurophysiology, they don’t provide significant conceptual insights into the LGN function.

We would like to thank the reviewer for the feedback. We agree that the paper provides a unique and powerful comparison between human and monkey neurophysiology. We now realize that we may have undersold the new conceptual insights into LGN function. We discovered unknown changes in spontaneous activity during eye closure, and cells that dramatically increase their activity when their preferred eye is shut. These findings have not been observed previously, and would be difficult, if not impossible, to study in animals. This new result is of great interest for scientists interested in the competition between the eyes (e.g. binocular rivalry and ocular gain control).

A major limitation is that a single subject provided the receptive field and tunning information, as the first patient provided coarse functional assessments with a flashing light. There were also a limited number of well-isolated neurons and many multi-unit responses showing intermediate effects. There are also no trial-by-trial roster plots showing within-subject response consistency.

We agree that the number of units is limited by the rarity of this surgical approach which precludes adding new patients. However, many of the results we present do not require large sample sizes, for example our results confirm the existence of red-green opponent cells in human LGN. We have also added the wave-shapes from patient #1 to our analysis of waveshape width to further increase the sample size and use of patient 1’s data. We have added raster-plots to Figure 3 to allow judgement of the reliability of the responses. Individual trial data is also shown in Figure 4.

Finally, given the sparse sampling and contentious role of the spike-wave shapes, assigning cell layers to unit responses might be premature.

We have added an extensive discussion of the assignment of cells to layers in the new extended Discussion section. Layer assignments were made by considering all measured tuning properties of the unit, i.e. the ocularity, preferred spatial and temporal frequency, and color-tuning. In addition, we considered the depth of the unit along the electrode trajectory and, in cases where the assignment was uncertain, we used the properties of other units on the same track. For example, Unit #5 could not be unambiguously assigned to a layer based on its RF characteristics, but units

located more dorsal and ventral to this unit were estimated to be located in layers 6 and 4, respectively. Furthermore, a multi-unit clustered from the same electrode at the same depth had ipsilateral dominant responses, making it very likely that unit #5 was in layer 5. We did not use the waveshape when assigning cells to layers.
