# Supplementary material for: Effects of eye closure on the spiking activity of human lateral geniculate neurons
Source: Nat Commun. 2025 Nov 24;16:10402. doi: 10.1038/s41467-025-65383-x (PMC12645034; doi:10.1038/s41467-025-65383-x)
Supplement: Supplementary file 4 — Transparent Peer Review file [file 41467_2025_65383_MOESM4_ESM.pdf]

Title: Supplementary Movie 1

Description: The video shows the online monitoring of spiking signals from Patient 2 during 8Hz photic stimulation (visible in the top-right corner of the video)
